# The burden of refraction disorders in 204 countries and territories from 1990 to 2021: A systematic analysis from the global burden of disease 2021

**DOI:** 10.1016/j.aopr.2024.11.001

**Published:** 2024-11-06

**Authors:** Yunhan Shen, Linyan Wang, Yuxin Cui, Bangxun Mao, Grace Loy Ming Hooi, Oluwatobi Idowu, Juan Ye, Tiansheng Zhu

**Affiliations:** a College of Mathematics and Computer Science, Zhejiang A & F University, Hangzhou, China; b Eye Center, The Second Affiliated Hospital of Zhejiang University School of Medicine, Zhejiang Provincial Key Laboratory of Ophthalmology, Zhejiang Provincial Clinical Research Center for Eye Diseases, Zhejiang Provincial Engineering Institute on Eye Diseases, Hangzhou, China; c Department of Ophthalmology, Affiliated Jinhua Hospital of Zhejiang University School of Medicine, Jinhua, China; d Department of Ophthalmology, Affiliated Lishui Hospital of Zhejiang University, Lishui, China; e Edinburgh Medical School, University of Edinburgh, Edinburgh, UK; f Weill Cornell Graduate School of Medical Sciences, New York, USA; g Guinness Eye Centre Lagos University Teaching Hospital Lagos, Lagos, Nigeria

**Keywords:** Refraction disorders, Global burden of disease, Public health

## Abstract

**Background:**

Refraction disorders are common eye conditions that cause blurred vision and, if left uncorrected, remain a leading cause of moderate to severe visual impairment worldwide. Despite this, a comprehensive assessment of the associated burden is lacking. This study aims to describe and predict the burden and regional distribution of refraction disorders using data from the 2021 Global Burden of Disease (GBD) study.

**Methods:**

This study utilized data from the Global Burden of Disease (GBD) 2021 on refraction disorders from 1990 to 2021. We analyzed the prevalence and disability-adjusted life years (DALYs) of refraction disorders and calculated the corresponding average annual percentage changes (AAPCs) in different gender and age groups across 204 countries and territories and 21 geographical regions. We employed the Bayesian Age-Period-Cohort (BAPC) model to predict the future burden of refraction disorders.

**Results:**

From 1990 to 2021, the global age-standardized prevalence rate (ASPR) of refraction disorders decreased from 2053.56 (per 100000, 95% Uncertainty Interval [UI]: 1835.31 to 2275.80) to 1919.66 (per 100000, 95%UI: 1715.24 to 2135.28), with an average annual percentage change (AAPC) of −0.21% (95% Confidence Interval [CI]: −0.23% to −0.19%). The age-standardized DALY (Disability-Adjusted Life Years) rate also declined from 88.04 (per 100000, 95%UI: 62.19 to 125.15) to 79.11 (per 100000, 95%UI: 54.94 to 114.14) with an AAPC of −0.33% (95%CI: −0.36% to −0.31%). Refraction disorders remain a significant burden in regions with lower Socio-demographic Index (SDI), particularly in parts of South Asia and Western Sub-Saharan Africa. Older individuals and females are disproportionately affected. The age-standardized DALY rate is expected to decline steadily before stabilising at approximately 77.94 per 100000 by 2030.

**Conclusions:**

From 1990 to 2021, the disease burden of refraction disorders showed a declining trend, but the decrease was not substantial. In some low-middle SDI regions, the burden remains high. Moreover, globally, women bear a higher burden than men. This study provides important information for the treatment and prevention of refraction disorders.

## 1 Introduction

Refraction disorder is a prevalent ocular condition that can cause blurred vision or even visual impairment.^1^^,^^2^ Despite being a central focus of the "VISION 2020: The Right to Sight" initiative spearheaded by the World Health Organization (WHO) and the International Agency for the Prevention of Blindness in 1999, uncorrected refraction disorder remains the leading cause of moderate to severe visual impairment worldwide and continues to impose a significant disease burden.^3^ In 2020, an estimated 295 (267–325) million people were living with moderate to severe visual impairment globally, and it is projected that by 2030, this number will increase to 474 (428–518) million people with moderate to severe visual impairment, with uncorrected refraction disorder being the leading cause of moderate to severe visual impairment worldwide.^4^

Past research has primarily focused on the pathological mechanisms and corrective treatments for refraction disorders,^5^^,^^6^ with relatively limited investigation into the overall trends and burden of these conditions.^7^^,^^8^ To address this gap, our study leverages the comprehensive data estimates provided by the Global Burden of Disease (GBD) 2021 study. This dataset includes global information on refraction disorders from 1990 to 2021, covering 204 countries and territories, 21 regions, and different genders and age groups.^9^ Our research aims to provide a detailed overview of the trends in refraction disorders across various demographics and regions, and to predict future burdens. By improving the understanding of these trends, we hope to offer more accurate and comprehensive information to support the prevention and control of refraction disorders.

## 2 Methods

### 2.1 Overview

The epidemiological data and estimations on refraction disorders utilized in this study were derived from the GBD 2021.^9^ The methodology employed in the GBD 2021 studies has been extensively described in existing literature.^10^, ^11^, ^12^ This study does not include any personal or medical information pertaining to identifiable living individuals, and no animal subjects were involved. The study adheres to the principles outlined in the revised 2013 Declaration of Helsinki.

### 2.2 Data sources

The data for this study is publicly available and obtained from the Global Health Data Exchange (GHDX) GBD Results Tool (http://ghdx.healthdata.org/gbd-results-tool). GBD 2021 utilized the latest available epidemiological data and improved standardized methods to comprehensively assess 371 diseases and injuries,^9^ 288 causes of death,^13^ and 88 risk factors,^14^ providing a comprehensive evaluation of health loss. The estimation process of GBD relies on identifying multiple relevant data sources for each disease or injury, including population censuses, household surveys, civil registration and vital statistics, disease registries, healthcare utilization, air pollution monitoring, satellite imaging, and disease reporting.^9^ We extracted the relevant prevalence, DALYs, and age-standardized rates (ASRs, per 100000 population) data for refraction disorders in 204 countries and territories from 1990 to 2021.

DALYs represent the sum of years lived with disability (YLDs) and years of life lost (YLLs).^9^ YLDs represent the number of individuals affected by a disease multiplied by disability weights, while YLLs represent the number of deaths multiplied by standard life expectancy loss. Since refraction disorders are not considered to result in death, YLLs were not estimated in the respective GBD study, and YLDs are approximately equivalent to DALYs. Age-standardized rates are estimated using the GBD world population age standard. By comparing the changes in age-standardized rates and the overall numbers, we can estimate the changes in prevalence and burden over time. All rates are reported per 100000 population.

### 2.3 Data prediction model

We employed the Bayesian Age-Period-Cohort (BAPC) model to project the burden of refraction disorders after 2021. The reliability of the BAPC model has been demonstrated in previous literature.^15^^,^ ^16^ The model utilized a Poisson model and divided the data by country, gender, and age, fitting it within the Integrated Nested Laplace Approximation (INLA) framework. Age, period, and/or cohort effects were modeled using either a second-order random walk (RW2) or fixed effect (drift). The BAPC model effectively avoids the complexity of Markov Chain Monte Carlo (MCMC) sampling, making the model easier to apply in practice, and provides well-calibrated probabilistic forecasts with prediction intervals that are not overly wide, outperforming the generalized Lee-Carter model.^17^ The analyses were conducted using the INLA and BAPC packages in R language (version 4.2.3), and all data analyses were performed using the open-source software R.

### 2.4 Statistical analysis

We conducted a secondary analysis of the prevalence and DALYs (disability-adjusted life years) of refraction disorders at the national and regional levels from 1990 to 2021. To better understand the relative burden of refraction disorders in different gender, age, year, and location groups, we utilized age-standardized prevalence rates (ASPRs) and age-standardized DALY rates in our research. To examine the global trends in refraction disorders, Joinpoint regression analysis was performed.^18^ By applying a logarithmic transformation and using the binomial approximation method, corresponding standard errors were obtained. The average annual percentage changes (AAPCs) of ASRs and their corresponding 95% confidence intervals (CI) were calculated using geometric weighted calculations based on values in population, gender, and age groups.^11^^,^^19^ The AAPCs and the lower bound of the confidence interval indicate an upward trend, while the AAPCs and the upper bound of the confidence interval indicate a downward trend.

In the overall study, we analyzed the association between SDI and the burden of refraction disorders using locally weighted regression. The study was conducted using R program (version 4.3.1, BAPC: version 4.2.3) and Joinpoint software (version 5.0).

## 3 Results

### 3.1 The burden and trends of refraction disorders worldwide from 1990 to 2021

**Fig. 1:**
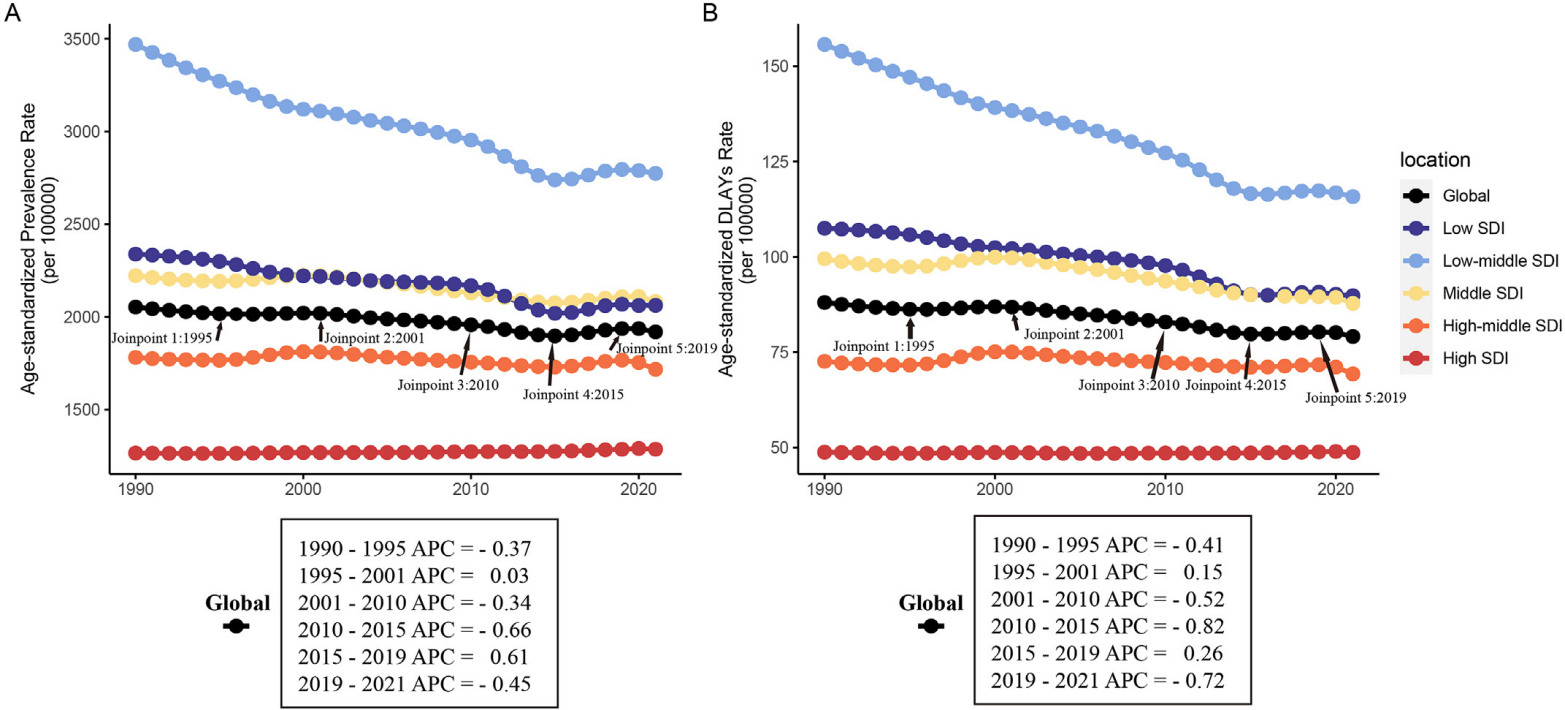
The burden of refraction disorders across different SDI quintiles from 1990 to 2021 and Joinpoint regression analysis. Age-standardized Prevalence(A) and DALY (B) rates of Refraction Disorder. DALY, disability-adjusted life year; SDI, Socio-demographic Index. APC, annual percentage changes.

We provide a comprehensive overview corresponding burden of refraction disorders at global and regional levels from 1990 to 2021 (Fig. 1, Table 1, Supplementary Table 1, and Supplementary Table 2). Globally, the ASPRs and age-standardized DALY rates of refraction disorders showed a slight decrease with AAPCs of −0.21 (−0.23 to −0.19) and −0.33 (−0.36 to −0.31) respectively, with changes of −6.52% (−7.25% to −5.82%) and −10.14% (−11.81% to −8.45%) (Table 1, and Supplementary Table 1). ASPRs have been consistently decreasing since 1990, with only a slight increase observed between 1995 and 2001 (APC ​= ​0.03) (Fig. 1A). However, there was a sharp rise between 2015 and 2019 (APC ​= ​0.61), followed by a steep decline between 2019 and 2021 (APC ​= ​−0.45). The age-standardized DALY rates exhibit a similar trend (Fig. 1B). They showed an increasing trend between 1995 and 2001 (APC ​= ​0.15) and between 2015 and 2019 (APC ​= ​0.26), followed by a rapid decline between 2019 and 2021 (APC ​= ​−0.72). The ASPRs of refraction disorders decreased from 2053.56 (per 100000, range: 1835.31 to 2275.80) in 1990 to 1919.66 (per 100000, range: 1715.24 to 2135.28) in 2021, and the age-standardized DALY rates decreased from 88.04 (per 100000, range: 62.19 to 125.15) in 1990 to 79.11 (per 100000, range: 54.94 to 114.14) in 2021(Table 1). However, both the prevalence cases and DALYs cases actually increased, with prevalence cases increasing from 95.98 (86.24–106.04) million in 1990 to 159.77 (142.53–178.70) million in 2021, and DALYs cases increasing from 4.03 (2.82–5.81) million in 1990 to 6.61 (4.60–9.53) million in 2021(Supplementary Table 2).

**Table 1:** Age-standardized rates of prevalence and DALY due to Refraction disorders in global and different regions, in 1990 and 2021, and their average annual percent changes from 1990 to 2021.

	Prevalence				DALYs			
	Age-standardized Prevalence rate per 100000 people (95%UI)		AAPC, 1990–2021 (95%CI)	*P* Value	Age-standardized DALY rate per 100000 people (95%UI)		AAPC, 1990–2021 (95%CI)	*P* Value
	1990	2021			1990	2021		
Global	2053.56 (1835.31–2275.8)	1919.66 (1715.24–2135.28)	−0.21 (−0.23 to −0.19)	<0.001	88.04 (62.19–125.15)	79.11 (54.94–114.14)	−0.33 (−0.36 to −0.31)	<0.001
**Sex**								
Female	2122.71 (1898.65–2359.66)	2010.3 (1795.38–2236.72)	−0.17 (−0.19 to −0.15)	<0.001	90.52 (63.93–128.48)	82.63 (57.5–118.94)	−0.28 (−0.31 to −0.26)	<0.001
Male	1988.64 (1779.77–2199.85)	1828.35 (1631.82–2032.97)	−0.26 (−0.28 to −0.24)	<0.001	85.73 (60.55–122.05)	75.52 (52.31–109.24)	−0.4 (−0.42 to −0.38)	<0.001
**SDI group**								
High SDI	1266.1 (1134.08–1396.02)	1286.91 (1153.4–1412.4)	0.06 (0.04–0.08)	<0.001	48.76 (32.95–71.39)	48.7 (32.87–71.33)	0 (−0.01 to 0.02)	0.822
High-middle SDI	1781.54 (1594–1976.58)	1717.63 (1535.02–1903.64)	−0.11 (−0.13 to −0.08)	<0.001	72.61 (50.9–104.27)	69.3 (47.99–100.28)	−0.13 (−0.18 to −0.09)	<0.001
Middle SDI	2222.7 (1989.98–2471.07)	2083.27 (1863.56–2310.25)	−0.2 (−0.22 to −0.17)	<0.001	99.52 (71.24–139.67)	87.81 (61.48–126.06)	−0.39 (−0.42 to −0.36)	<0.001
Low-middle SDI	3469.56 (3090.76–3878.16)	2774.05 (2453.7–3115.98)	−0.71 (−0.75 to −0.68)	<0.001	155.73 (110.68–218.15)	115.82 (80.26–165.83)	−0.95 (−0.98 to −0.92)	<0.001
Low SDI	2338.79 (2086.21–2609.68)	2062.51 (1826.09–2328.2)	−0.41 (−0.45 to −0.37)	<0.001	107.51 (77.31–149.99)	89.83 (62.97–126.44)	−0.58 (−0.62 to −0.54)	<0.001
**Geographic regions**								
Andean Latin America	2733.78 (2448.24–3024.76)	2642.66 (2381.51–2927.88)	−0.12 (−0.16 to −0.09)	<0.001	113.29 (78.18–164.54)	103.92 (70.64–151.08)	−0.29 (−0.32 to −0.25)	<0.001
Australasia	1372.15 (1232.94–1524.83)	1370.99 (1223.5–1526.15)	0 (−0.06 to 0.06)	0.922	50.09 (34.07–74.3)	49.73 (33.15–73.88)	−0.01 (−0.07 to 0.05)	0.674
Caribbean	1831.48 (1639.68–2029.86)	1717.08 (1531.96–1909.93)	−0.2 (−0.21 to −0.2)	<0.001	72.39 (49.28–106.48)	65.96 (44.48–97.75)	−0.29 (−0.31 to −0.28)	<0.001
Central Asia	1826.84 (1619.37–2049.87)	1746.41 (1546.44–1964.81)	−0.14 (−0.15 to −0.14)	<0.001	69.16 (46.84–101.42)	65.59 (44.25–97.47)	−0.17 (−0.19 to −0.16)	<0.001
Central Europe	1282.96 (1137.58–1436.73)	1259.82 (1114.76–1410.88)	−0.05 (−0.07 to −0.04)	<0.001	45.19 (29.83–67)	44.5 (29.49–65.52)	−0.04 (−0.08 to 0)	0.031
Central Latin America	2412.28 (2160.22–2667.54)	2226.69 (1990.96–2459.71)	−0.25 (−0.28 to −0.22)	<0.001	106.26 (74.75–152.23)	93.6 (65.18–135.81)	−0.41 (−0.42 to −0.39)	<0.001
Central Sub-Saharan Africa	1706.33 (1515.21–1921.43)	1741.79 (1548.33–1952.78)	0.06 (0.05–0.08)	<0.001	63.79 (43.85–92.54)	64.98 (44.13–94.29)	0.06 (0.04–0.07)	<0.001
East Asia	1557.41 (1387.6–1731.54)	1456.79 (1291.02–1629.15)	−0.18 (−0.23 to −0.14)	<0.001	73.35 (53.26–100.85)	64.98 (46–91.1)	−0.35 (−0.41 to −0.28)	<0.001
Eastern Europe	2241.99 (2007.75–2503.35)	2188.17 (1961.21–2444.5)	−0.08 (−0.09 to −0.07)	<0.001	81.17 (54.56–120.6)	78.06 (52.35–115.98)	−0.13 (−0.14 to −0.12)	<0.001
Eastern Sub-Saharan Africa	1121.13 (1014.05–1235.77)	1068.75 (958.13–1188.04)	−0.16 (−0.18 to −0.13)	<0.001	56.42 (41.36–77.58)	51.29 (36.68–71.99)	−0.31 (−0.32 to −0.29)	<0.001
High-income Asia Pacific	1189.5 (1071.02–1307.36)	1192.53 (1070.1–1310.75)	0.01 (−0.01 to 0.02)	0.317	46.26 (31.31–67.56)	45.78 (31.03–67.33)	−0.03 (−0.05 to −0.01)	0.005
High-income North America	1051.17 (935.58–1165.51)	1065.69 (950.02–1181.34)	0.04 (0.02–0.06)	<0.001	39.97 (26.92–58.58)	39.69 (26.6–58.45)	−0.03 (−0.04 to −0.01)	0.002
North Africa and Middle East	2386.92 (2147.7–2636.84)	2280.95 (2040.24–2514.18)	−0.15 (−0.17 to −0.13)	<0.001	106.87 (75.37–151.65)	97.01 (67.72–139.63)	−0.32 (−0.34 to −0.29)	<0.001
Oceania	2595.06 (2253.14–2965.26)	2518.86 (2183.35–2876.59)	−0.11 (−0.15 to −0.07)	<0.001	83.02 (53.33–126.54)	79.66 (50.59–121.23)	−0.14 (−0.17 to −0.11)	<0.001
South Asia	4446.18 (3962.59–5006.29)	3397.67 (2991.52–3859.72)	−0.87 (−0.91 to −0.83)	<0.001	201.54 (144.62–281.61)	141.73 (98.52–201.42)	−1.14 (−1.21 to −1.07)	<0.001
Southeast Asia	1838.75 (1644.85–2042.66)	1633.62 (1470.42–1800.88)	−0.39 (−0.43 to −0.35)	<0.001	77.89 (53.77–111.99)	65.31 (44.11–95.59)	−0.58 (−0.61 to −0.54)	<0.001
Southern Latin America	1978.74 (1782.79–2187.75)	1893.73 (1692.83–2098.87)	−0.14 (−0.16 to −0.13)	<0.001	74.15 (49.72–109.58)	68.76 (45.66–101.75)	−0.24 (−0.26 to −0.23)	<0.001
Southern Sub-Saharan Africa	1568.84 (1402.08–1742.35)	1535.31 (1361.35–1716.51)	−0.05 (−0.12 to 0.01)	0.1	70.29 (50.26–97.95)	66.2 (46.92–93.48)	−0.18 (−0.23 to −0.13)	<0.001
Tropical Latin America	2968.45 (2679.31–3262.59)	2810.82 (2524.81–3085.81)	−0.16 (−0.27 to −0.05)	0.005	122.13 (84.37–177.31)	112.22 (77–163.19)	−0.26 (−0.36 to −0.16)	<0.001
Western Europe	1600.58 (1437.43–1763.41)	1564.83 (1401.07–1733.45)	−0.07 (−0.09 to −0.06)	<0.001	61.9 (42.03–90.52)	59.23 (39.76–87.31)	−0.14 (−0.15 to −0.13)	<0.001
Western Sub-Saharan Africa	1199.13 (1073.99–1329.43)	1350.54 (1202.95–1519.49)	0.38 (0.34–0.41)	<0.001	57.78 (41.61–80.8)	63.29 (44.79–89.46)	0.29 (0.27–0.31)	<0.001

DALYs, disability adjusted life years; UI, uncertainty interval; SDI, Socio-demographic Index; AAPC: average annual percent change; CI: Confidence Interval.

Among the five SDI regions (Fig. 1), the Low-middle SDI region had the highest ASPRs (2774.05 per 100000, range: 2453.70 to 3115.98) and age-standardized DALY rates (115.82 per 100000, range: 80.26 to 165.83), but showed the most significant downward trend (AAPC of ASPRs: −0.71, range: −0.75 to −0.68; AAPC of age-standardized DALY rates: −0.95, range: −0.98 to −0.92). The Middle SDI region had the highest actual burden in 2021 in terms of prevalence cases (54.54 million, range: 48.75 to 61.05 million) and DALYs cases (2.31 million, range: 1.61 to 3.30 million). Among all the regions, only the High SDI region showed a slight increase in ASPRs (AAPC: 0.06, range: 0.04 to 0.08), while the other indicators showed a downward trend.

Among the 21 geographic regions, the age-standardized DALY rate and ASPR in most geographical areas have shown a downward trend over the past 30 years (Supplementary Fig. 1 and Table 1), South Asia had the highest ASPRs (3397.69 per 100000, range: 2991.52 to 3859.72) and age-standardized DALY rates (141.73 per 100000, range: 98.52 to 201.42). In terms of ASPRs trend, South Asia had the most significant decrease (AAPC: −0.87, range: −0.91 to −0.83), while Western Sub-Saharan Africa had the most significant increase (AAPC: 0.38, range: 0.34 to 0.41). Additionally, South Asia (AAPC: −1.14, range: −1.21 to −1.07) and Western Sub-Saharan Africa (AAPC: 0.29, range: 0.27 to 0.31) were also the two regions with the most significant increase and decrease respectively in age-standardized DALY rates.

### 3.2 Correlations between SDI and the burden and trends of refraction disorders

**Fig. 2:**
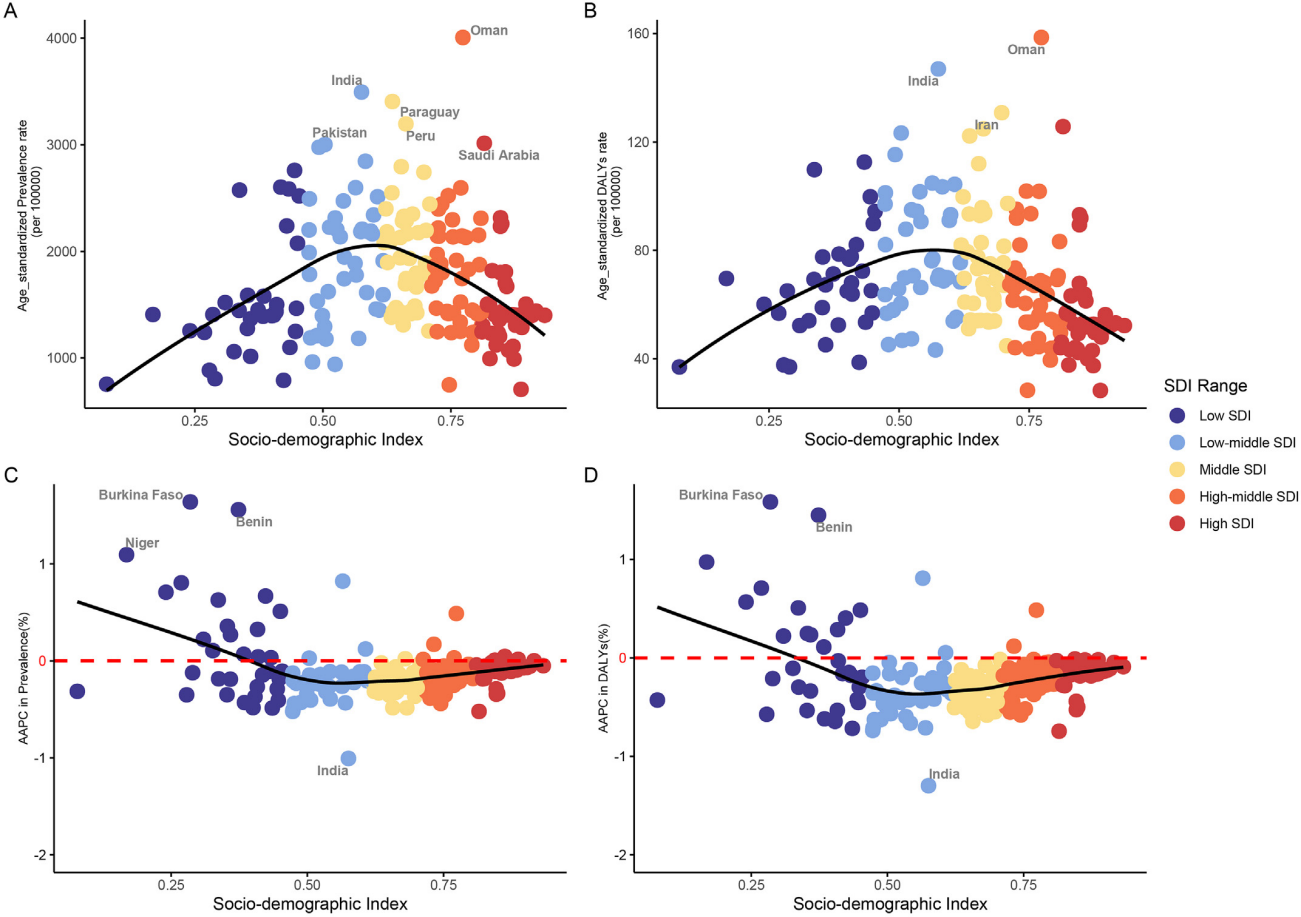
In 2021, the burden of refraction disorders, AAPCs from 1990 to 2021, and their association with SDI across countries and territories. Age-standardized prevalence (A) and DALY (B) rates of refraction disorder. AAPCs in Age-standardized prevalence(C) and DALY(D) rates between 1990 and 2021. DALY, disability-adjusted life year; SDI, Socio-demographic Index; AAPC, average annual percentage changes.

A U-shaped relationship was found between the ASPRs (Fig. 2A) and age-standardized DALY rates (Fig. 2B) of refraction disorders and SDI in 2021. In regions with lower and higher SDI, the ASPRs and age-standardized DALY rates of refraction disorders are relatively low, while the burden is higher in regions closer to the Low-middle SDI and Middle SDI (Fig. 2A and B). Among the 21 geographic regions, the burden of refraction disorders in 2021 also demonstrates a U-shaped relationship with SDI (Supplementary Table 1). From 1990 to 2021, the AAPCs of ASPRs and age-standardized DALY rates exhibit a U-shaped relationship with SDI. In most regions with lower SDI, the AAPCs are greater than 0, indicating an increasing burden, while in other regions, the AAPCs are generally below 0, indicating a decreasing trend (Fig. 2C and D).

### 3.3 Correlations between 204 countries and territories and burden and trends of refraction disorders

Several countries in the Indian Ocean coast and Southeast Asia region exhibit higher burdens of refraction disorders. Globally, the burden of refraction disorders has shown a decrease in most regions and countries, with significant declines observed in some high-burden areas. However, certain countries in the central sub-Saharan Africa region have experienced an increasing trend. Additionally, there has been an increase in the burden of refraction disorders in some high-income countries. Oman (4007.19 per 100000), India (3495.70 per 100000), and Paraguay (3407.02 per 100000) have the highest ASPRs among countries and territories, while Barbados (718.96 per 100000), Burundi (783.80 per 100000), and Uganda (788.68 per 100000) have the lowest ASPRs (Supplementary Table 3). In 2021, ASPRs in Oman, India, Paraguay, Pakistan, Peru, Saudi, and Arabia exceeded 3000 per 100000. The three countries/territories with the highest age-standardized DALY rates are Oman (158.56 per 100000), India (146.97 per 100000), and Iran (130.81 per 100000) (Supplementary Table 3). From 1990 to 2021, the AAPCs in ASPRs were greater than 0 in 29 countries and territories, indicating an increasing trend. The three countries/territories with the highest AAPCs are Cote d'Ivoire (2.28), Burkina Faso (1.64), and Benin (1.56), while India (−1.01), Bhutan (−0.52), and Saudi Arabia (−0.52) showed the most significant decreases among the countries/territories with AAPCs less than 0 (Supplementary Table 3). For age-standardized DALY rates, 18 countries and territories had AAPCs greater than 0, indicating an upward trend. The three countries/territories with the highest AAPCs are Cote d'Ivoire (2.36), Burkina Faso (1.58), and Benin (1.45), while India (−1.30), Saudi Arabia (−0.74), and Bhutan (−0.74) showed the most substantial decreases among the countries/territories with negative AAPCs (Supplementary Table 3).

### 3.4 Burden of refraction disorders in different sexes and age groups

**Fig. 3:**
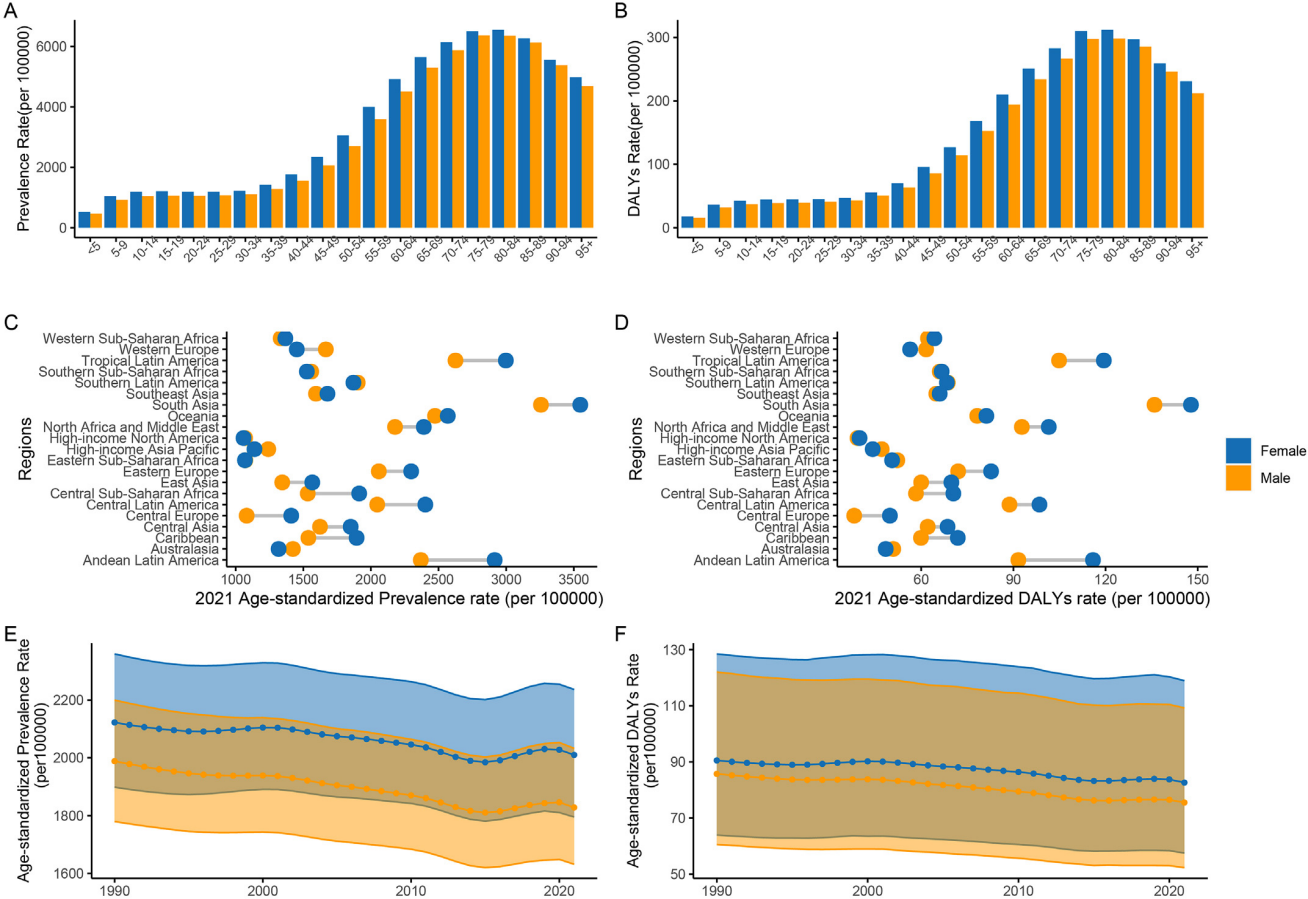
The burden of refraction disorder in different sexes and age groups in 2021 and change in different genders in 1990 and 2021. Prevalence (A) and DALY (B) rates of refraction disorder in different sexes and age groups. Age-standardized prevalence (C) and DALY (D) rates of refraction disorder in different sexes and geographic regions. Age-standardized prevalence(E) and DALY (F) rates of refraction disorder change in different genders in 1990 and 2021. DALY, disability-adjusted life year.

From 1990 to 2021, females consistently bore a heavier burden of refraction disorders compared to males. Both ASPRs (AAPC: Female −0.17; Male −0.26) and age-standardized DALY rates (AAPC: Female −0.28; Male −0.40) for refraction disorders showed a decreasing trend for both genders over the past three decades (Table 1, Fig. 3E and F). However, the actual burden of refraction disorders has been increasing over the years (Supplementary Fig. 2). This gender-based discrepancy was especially pronounced in regions with higher overall burdens, such as Tropical Latin America, South Asia, and Andean Latin America. (Fig. 3C and. D). The burden of refraction disorders also increases with age, and across all age groups, the burden on females is higher than that on males (Fig. 3A and B). Female ASPRs for refraction disorders reached their peak in the 80–84 years age group (6546.71 per 100000, range: 5174.30 to 8326.84), while for males, it peaked in the 75–79 years age group (6360.51 per 100000, range: 5051.06 to 7761.92). (Supplementary Table 4 and Fig. 3A). The age-standardized DALY rates for refraction disorders also peaked in the 80–84 years age group (Female: 311.92 per 100000; Male: 298.05 per 100000) (Supplementary Table 4 and Fig. 3B).

### 3.5 The future trends of refraction disorders predicted by BAPC

**Fig. 4:**
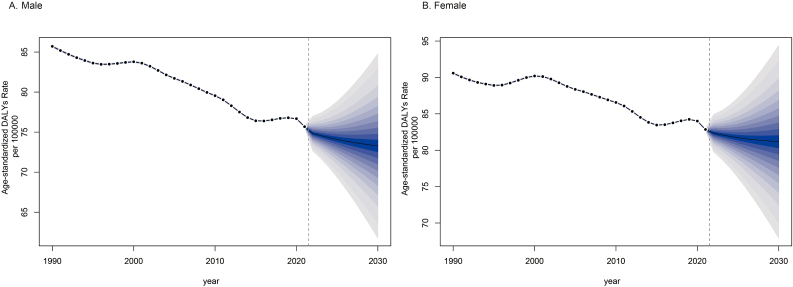
Trends in age-standardized DALY rates from 2021 to 2030 in males (A) and females (B) predicted by Bayesian age–period–cohort (BAPC) models.

According to the BAPC model, we have made corresponding predictions for the burden of refraction disorders in the future. The DALYs burden for both males and females has generally shown a decreasing trend over the past 30 years, but experienced a slight increase in 2015. Based on the BAPC model, the age-standardized DALY rates for refraction disorders are expected to continue to decline. The projected DALY cases for males in 2030 are estimated to be 3.48 million, with an age-standardized DALY rate of 73.27 per 100000 (Fig. 4A). For females, the projected DALY cases in 2030 are estimated to be 4.09 million, with an age-standardized DALY rate of 81.16 per 100000 (Fig. 4B).

## 4 Discussion

The global burden of refraction disorders is significant, affecting an estimated 2.2 billion individuals worldwide, with 1 billion cases deemed preventable.^20^ Our research contributes to this understanding by highlighting a declining trend in both prevalence and Disability-Adjusted Life Years (DALY) rates, with AAPCs of −0.21 and −0.33, respectively, consistent with previous Global Burden of Disease (GBD) studies.^8^ Research shows that from 2000 to 2021, the effective refractive error coverage (eREC) increased by 19%, indicating that the effective coverage of vision correction services has significantly improved over the past 20 years.^7^ Due to the rapid global population growth and the further exacerbation of aging trends, the relative outcomes produced by the age-standardized rates have shown a declining trend over the past thirty years. However, the actual numbers caused by DALY and prevalence have increased from 1990 to 2021. This decline may be attributable to advancements in eye care services, increased access to corrective measures like eyeglasses or contact lenses, and the effectiveness of public health interventions aimed at promoting eye health.^21^ However, the persistent gap between the declining trend in age-standardized prevalence and DALY rates and the persistently high overall prevalence of refraction disorders suggests ongoing challenges, such as population growth and ageing demographics.^21^ The COVID-19 pandemic has further influenced these trends. The shift to online learning and increased screen time, combined with reduced outdoor activities due to home confinement, has led to a myopic shift, particularly among children.^22^, ^23^, ^24^ Interestingly, in our study, the APCs for ASPR and age-standardized DALY rate from 2019 to 2021 were −0.45 and −0.72, respectively, showing a decreasing trend. We speculate that the pandemic has underscored the importance of health, leading more people to prioritize their eye health, which could increase the number of regular check-ups and early detection of refraction disorders. Overall, while there are signs of progress in addressing refraction disorders, continued investment in eye care services to further reduce the burden of refraction disorders globally is required.

Refraction disorders are caused by a combination of genetic and environmental factors. A previous study indicated that retinal factors, including retinal images and dopamine levels, as well as optical factors such as pupil size and peripheral retinal defocus, may influence the occurrence and development of myopia by regulating the process of eye growth.^25^ Moreover, environmental factors such as the structure of the visual environment, outdoor time, and light levels may affect the transmission of visual signals to the retina, thereby regulating the growth and development of the eyeball, which in turn leads to the onset and progression of myopia.^26^^,^^27^ In our study, it was found that the burden of refractive disorders in South Asia is significantly higher than in other regions. A previous study found that the high prevalence of myopia in the Asian region is due to the intensive education system and the lack of outdoor activity time.^28^ Another study based on the European population also explained that an increase in education levels would lead to a rise in the incidence of myopia.^29^ Another study also demonstrated a correlation between outdoor activity time and the risk of myopia in adolescents and children, and increasing outdoor activity by 1 ​h per week can reduce the likelihood of myopia by 2%.^30^ Meanwhile, the effective refractive error coverage (eREC) for adults aged 50 and above in South Asia is only 9.0%, indicating that the region lacks sufficient refractive correction services and that there is a deficiency in eye care services.^7^ Additionally, we also observed substantial burdens in North Africa, the Middle East, and Oceania. The eREC in North Africa and the Middle East is relatively high (62.1%), while in Southeast Asia, East Asia, and Oceania, it is comparatively lower (40.0%).^7^ These three regions still have some gaps in providing effective refractive error correction services and need to further enhance the coverage of these services. Factors such as genetic predispositions, environmental influences, and cultural practices might contribute to these regional disparities.^6^^,^^31^ Regional disparities in refraction disorders are notable, with low to middle SDI regions exhibiting a higher disease burden compared to high SDI areas. This discrepancy is likely due to differential investment in refraction disorder correction, heightened health awareness, and enhanced access to healthcare systems.^32^ This is also reflected in the effective refractive error correction coverage (eREC), where the eREC in high-income regions was 74.1% (95% CI: 67.5%–79.7%) in 2021, compared to only 13.6% (95%CI: 12.4%–14.8%) in low-income regions, indicating a significantly higher eREC in high-income areas compared to low-income areas.^7^ In some high SDI areas, there may be greater investment in the correction of refraction disorders, leading to a lower burden. In some developing countries, the lack of trained personnel and a comprehensive system for refractive examinations contribute to the high prevalence of refraction disorders in low SDI countries.^33^ In many low SDI countries, the AAPCs is greater than 0, indicating an increasing trend. We speculate that the lack of corresponding improvements in healthcare despite the widespread education and improvements in living standards in low SDI countries is a significant factor contributing to this phenomenon. Multiple studies have shown an association between the level and duration of education and refraction disorders.^6^^,^^34^ The increasing use of smartphones may also be a contributing factor to the rising prevalence of refraction disorders in low SDI countries.^35^ Additionally, correcting refraction disorders incurs varying costs, with higher expenses observed in European regions. Additionally, correcting refraction disorders incurs varying costs, with higher expenses observed in European regions.^33^ Socioeconomic barriers such as elevated treatment costs, insufficiently trained personnel, and inadequate healthcare infrastructure contribute significantly to refraction disorder prevalence, particularly in the low to middle SDI regions. Addressing these systemic challenges within the framework of universal health coverage is paramount to enhancing access and equity in eye care services globally. Take China as an example. In 2018, the country launched a comprehensive national program to address the issue of uncorrected refractive errors among children.^36^ This program focuses on school-based prevention, screening, and treatment, encourages parental involvement, and advocates for reducing harmful behaviors such as excessive screen use. According to monitoring data from the China CDC, by 2022, the overall myopia rate among children and adolescents in China had reached 51.9%, with a breakdown of 36.7% in primary school, 71.4% in middle school, and 81.2% in high school.^37^ Although the situation remains severe, there has been a slight decrease of 0.7 percentage points compared to the 2021 rate of 52.6%. More notably, compared to the baseline survey in 2018, which recorded a rate of 53.6%, the overall myopia rate has decreased by 1.7 percentage points. Among students who are already myopic, the proportions of mild, moderate, and high myopia are 53.3%, 37.0%, and 9.7%, respectively, with a decrease in the proportion of high myopia.^37^ These data indicate significant positive outcomes from China's efforts in myopia prevention and control. This is also reflected in our study, where the prevalence of refractive disorders in China shows a decreasing trend, with an AAPC of −0.17% for prevalence and −0.34% for DALYs (Supplementary Table 3). In conclusion, a multifaceted approach considering socio-demographic factors, healthcare infrastructure, and educational accessibility is essential for informing targeted public health interventions and policy decisions to alleviate the global burden of refraction disorders.

Systemic inequalities in age and gender have consistently influenced the burden of refraction disorders. Over the past three decades, females have been more vulnerable to refraction disorders compared to males, with age and socioeconomic levels serving as persistent confounding variables. At younger ages, females have a higher probability of experiencing refraction disorders, which can be attributed to the fact that females undergo puberty earlier than males.^38^^,^^39^ Among the elderly population, gender disparities persist, potentially due to economic and social inequalities.^40^ Given the longer life expectancy of women and existing gender inequalities in healthcare access, it is imperative to place greater emphasis on supporting the visual health of elderly women while ensuring equitable treatment for all patients regardless of gender. Addressing these disparities is crucial for ensuring equitable eye care and improving overall health outcomes.

In this study, we used the BAPC model to investigate the future burden of refraction disorders. Compared to other models, the BAPC model is able to more accurately predict and reflect the future burden of diseases in global burden of disease research.^17^ Our findings indicate a continued decline in refraction disorders before plateauing in 2030. Persistent age and gender disparities are anticipated, with the burden still disproportionately affecting the elderly and female populations. However, the BAPC model is subject to inherent uncertainties and methodological limitations. It relies on historical data and the assumption that past trends will persist, which may not always hold due to evolving healthcare landscapes, socioeconomic dynamics, and unexpected events such as the COVID-19 pandemic. Therefore, it is essential to interpret the results cautiously and consider them as reference points rather than definitive conclusions.

Although this study provides a comprehensive analysis of the burden of refraction disorders in 204 countries and regions worldwide, considering factors such as age and gender. However, several limitations should be noted. Firstly, this study is fundamentally an observational analysis, and it cannot determine whether there is a causal relationship between the burden of refractive errors and the other observed factors. Although differences in age and gender were emphasized, the underlying causes and potential intervention measures were not thoroughly explored. Secondly, the study lacks detailed information on specific risk factors and the extent of visual impairment, limiting insights into the impact on quality of life and productivity. Thirdly, GBD data may inherently contain inaccuracies and inconsistencies during its collection and reporting processes, especially in high-risk regions where the burden might be underestimated. Additionally, the research did not delve into specific high-prevalence areas, limiting its scope. Future research should address these gaps and develop targeted prevention and treatment strategies for a more comprehensive understanding of refraction disorders globally.

## 5 Conclusions

From 1990 to 2021, although the burden of refraction disorders showed a declining trend, the decrease was not substantial. Certain low-middle SDI regions, particularly South Asia, North Africa, and the Middle East continue to experience a high burden of this disease. Globally, women bear a higher burden of refraction disorders compared to men. This study provides a comprehensive analysis of refraction disorders over the past 30 years and offers corresponding predictions for the future burden. These insights are significant for informing further research, as well as the development of effective prevention and treatment strategies for refraction disorders.

## Study approval

Not Applicable.

## Author contributions

The authors confirm contribution to the paper as follows: TZ and JY:Conceptualization, Supervision, Funding acquisition, Writing-review & editing; YS: Data Curation, Writing-original draft preparation, Writing-review & editing, Visualization, Software; LW: Writing-original draft preparation, Writing-review & editing, Funding acquisition; YC, BM, GH and OI: Writing-review & editing. All authors reviewed the results and approved the final version of the manuscript.

## Declaration of generative AI in scientific writing

During the preparation of this work the author(s) used ChatGPT in order to polish the language of the initial draft for better readability. After using this tool/service, the author(s) reviewed and edited the content as needed and take(s) full responsibility for the content of the publication.

## Funding

The study was funded by the Applied Research of Public Welfare Technology of Zhejiang Province (LGF22H120007), National Natural Science Foundation Regional Innovation and Development Joint Fund(U20A20386), National Natural Science Foundation of China (82330032), the grant from startup found of Zhejiang A & F University under Grant (203402007101).

## Declaration of competing interest

The authors declare the following personal relationships which may be considered as potential competing interests: Oluwatobi O. Idowu is an employee of Genentech Inc. No conflict of interest for other authors.

## Abbreviations

DALYs: Disability-adjusted life years
SDI: Socio-demographic Index
ASR: Age-standardized rate
ASPR: age-standardized prevalence rate
AAPC: average annual percentage change
BAPC: Bayesian age-period-cohort
UI: Uncertainty interval
CI: Confidence interval
