# Supplementary material for: The burden of refraction disorders in 204 countries and territories from 1990 to 2021: A systematic analysis from the global burden of disease 2021
Source: Adv Ophthalmol Pract Res. 2024 Nov 6;5(2):79–87. doi: 10.1016/j.aopr.2024.11.001 (PMC11930593; doi:10.1016/j.aopr.2024.11.001)
Supplement: Multimedia component 1 [file mmc1.docx]

Supplementary Table 1. The percentage changes of refraction disorders in different countries and regions from 1990 to 2021

| location | Percentage change, % (95% UI) | | | |
| --- | --- | --- | --- | --- |
|  | Age-standardized Prevalence rate  per 100,000 people | Age-standardized Prevalence rate  per 100,000 people | Prevalence case | DALYs case |
| Global | -6.52 (-7.25 to -5.82) | -10.14 (-11.81 to -8.45) | 66.46  (62.25 to 70.69) | 64.27  (60.55 to 68.02) |
| High SDI | 1.64 (0.84 to 2.41) | -0.13 (-1.29 to 1) | 40.14  (35.47 to 45.27) | 41.43  (37.1 to 45.86) |
| High-middle SDI | -3.59 (-4.83 to -2.36) | -4.56 (-5.91 to -3.28) | 48.79  (43.91 to 53.33) | 51.73  (47.24 to 56.56) |
| Middle SDI | -6.27 (-7.37 to -5.29) | -11.77 (-14.12 to -9.49) | 79.12  (72.54 to 85.67) | 76.87  (71.35 to 82.65) |
| Low-middle SDI | -20.05 (-21.24 to -18.99) | -25.63 (-28.04 to -23.27) | 65.2  (61.82 to 68.82) | 58.33  (54.1 to 62.34) |
| Low SDI | -11.81 (-13.37 to -10.05) | -16.45 (-18.83 to -13.88) | 105.4  (102.27 to 108.75) | 97.54  (92.77 to 102.44) |
| Andean Latin America | -3.33 (-5.87 to -0.75) | -8.27 (-11.69 to -5.27) | 99.68  (91.74 to 108.41) | 97.07  (89.47 to 104.24) |
| Australasia | -0.08 (-2.84 to 2.72) | -0.71 (-4.78 to 3.38) | 62.02  (54.49 to 69.29) | 65.67  (56.13 to 75.52) |
| Caribbean | -6.25 (-8.02 to -4.72) | -8.89 (-11.17 to -6.6) | 43.24  (38.39 to 48.02) | 43.69  (38.53 to 48.72) |
| Central Asia | -4.4 (-6.14 to -2.67) | -5.17 (-7.19 to -3.24) | 49.75  (45.32 to 54.37) | 49.74  (45.07 to 54.28) |
| Central Europe | -1.8 (-3.08 to -0.67) | -1.54 (-3.07 to -0.14) | 15.95  (11.88 to 20.01) | 16.62  (12.33 to 21.34) |
| Central Latin America | -7.69 (-8.68 to -6.57) | -11.92 (-13.87 to -9.84) | 80.71  (73.82 to 87.65) | 82.58  (76.11 to 89.22) |
| Central Sub-Saharan Africa | 2.08 (-0.8 to 5.2) | 1.87 (-1.24 to 5.12) | 160.37  (153.45 to 168.54) | 160.53  (151.79 to 169.96) |
| East Asia | -6.46 (-8.12 to -4.66) | -11.41 (-14.03 to -8.68) | 80.79  (71.93 to 88.86) | 78.49  (71.2 to 85.36) |
| Eastern Europe | -2.4 (-3.42 to -1.35) | -3.83 (-5.26 to -2.46) | 5.28  (2.87 to 7.57) | 4.09  (1.85 to 6.42) |
| Eastern Sub-Saharan Africa | -4.67 (-6.63 to -2.95) | -9.1 (-11.58 to -6.63) | 125.9  (121.73 to 129.9) | 118.87  (112.9 to 125.02) |
| High-income Asia Pacific | 0.25 (-0.97 to 1.31) | -1.05 (-2.93 to 0.82) | 30.45  (22.92 to 38.86) | 35.95  (27.63 to 45.42) |
| High-income North America | 1.38 (0.38 to 2.35) | -0.7 (-2.18 to 0.73) | 38.94  (34.95 to 43.32) | 39.59  (35.25 to 43.85) |
| North Africa and Middle East | -4.44 (-6.09 to -2.77) | -9.22 (-11.77 to -6.75) | 97.01  (90.46 to 103.7) | 95.07  (89.28 to 101.72) |
| Oceania | -2.94 (-5.67 to 0.09) | -4.05 (-7.16 to -0.86) | 129.9  (123.32 to 137.71) | 126.88  (118.48 to 134.87) |
| South Asia | -23.58 (-24.75 to -22.54) | -29.68 (-32.16 to -27.05) | 72.95  (68.92 to 77.32) | 64.19  (59.33 to 68.93) |
| Southeast Asia | -11.16 (-13.19 to -9.34) | -16.15 (-18.85 to -13.6) | 57.7  (52.8 to 62.85) | 56.72  (52.01 to 61.54) |
| Southern Latin America | -4.3 (-6.56 to -1.89) | -7.27 (-10.58 to -3.92) | 40.93  (36.42 to 45.56) | 39.7  (34.79 to 45.26) |
| Southern Sub-Saharan Africa | -2.14 (-3.6 to -0.76) | -5.82 (-7.88 to -4.02) | 75.82  (70.83 to 81.27) | 74.14  (69.39 to 79.27) |
| Tropical Latin America | -5.31 (-6.28 to -4.23) | -8.11 (-9.94 to -6.57) | 66.16  (59.64 to 73.16) | 69.59  (63.19 to 76.37) |
| Western Europe | -2.23 (-3.19 to -1.26) | -4.32 (-6.06 to -2.82) | 20.11  (16.4 to 24.03) | 20.78  (17.12 to 24.76) |
| Western Sub-Saharan Africa | 12.63 (10.37 to 15.04) | 9.53 (6.69 to 12.11) | 171.95  (167.06 to 176.8) | 165.57  (158.41 to 171.55) |

DALYs, disability adjusted life years; UI, uncertainty interval; SDI, Socio-demographic Index.
