# Supplementary material for: The burden of refraction disorders in 204 countries and territories from 1990 to 2021: A systematic analysis from the global burden of disease 2021
Source: Adv Ophthalmol Pract Res. 2024 Nov 6;5(2):79–87. doi: 10.1016/j.aopr.2024.11.001 (PMC11930593; doi:10.1016/j.aopr.2024.11.001)
Supplement: Multimedia component 2 [file mmc2.docx]

Supplementary Table 2. Prevalent cases and DALYs due to due to Refraction disorders in global and different regions, in 1990 and 2021.

| location | Prevalence case(95%UI) | | DALY case(95%UI) | |
| --- | --- | --- | --- | --- |
|  | 1990 | 2021 | 1990 | 2021 |
| Global | 95978319  (86236197 to 106044403) | 159765917  (142526915 to 178698348) | 4029084  (2821217 to 5812174) | 6618600  (4599082 to 9528676) |
| High SDI | 11796392  (10574699 to 12980520) | 16532003  (14744586 to 18239386) | 460957  (314571 to 669985) | 651929  (453966 to 929362) |
| High-middle SDI | 18429393  (16515827 to 20423165) | 27421792  (24305264 to 30943682) | 748735  (523143 to 1078997) | 1136033  (792645 to 1626121) |
| Middle SDI | 30450445  (27410659 to 33726613) | 54543247  (48754426 to 61052227) | 1306760  (908852 to 1882212) | 2311240  (1613507 to 3308195) |
| Low-middle SDI | 27847244  (24958860 to 30907719) | 46004885  (40990725 to 51552083) | 1191346  (834617 to 1705275) | 1886272  (1297734 to 2736015) |
| Low SDI | 7378532  (6606296 to 8197508) | 15155638  (13499747 to 16856286) | 318437  (222737 to 452158) | 629029  (435086 to 910576) |
| Andean Latin America | 857004  (769940 to 947111) | 1711240  (1539436 to 1893867) | 33925  (22875 to 49853) | 66855  (45187 to 97656) |
| Australasia | 283175  (254230 to 311935) | 458789  (408260 to 506019) | 10426  (7097 to 15435) | 17272  (11889 to 25063) |
| Caribbean | 596665  (531840 to 662766) | 854693  (761320 to 954030) | 23031  (15497 to 34081) | 33093  (22425 to 49070) |
| Central Asia | 1068170  (955419 to 1188286) | 1599533  (1409713 to 1804634) | 39882  (26628 to 59063) | 59718  (40061 to 88841) |
| Central Europe | 1763264  (1554068 to 1997193) | 2044563  (1782705 to 2326908) | 62153  (41054 to 91617) | 72485  (48717 to 105660) |
| Central Latin America | 3173170  (2840150 to 3534416) | 5734174  (5118445 to 6344218) | 131636  (90119 to 189569) | 240347  (166816 to 349851) |
| Central Sub-Saharan Africa | 534501  (473470 to 600708) | 1391685  (1239630 to 1546248) | 19134  (12715 to 28438) | 49849  (32874 to 74546) |
| East Asia | 15325817  (13678032 to 17105161) | 27707612  (24235005 to 31689353) | 708410  (507328 to 982029) | 1264468  (893967 to 1758611) |
| Eastern Europe | 5691994  (5078235 to 6394207) | 5992338  (5304238 to 6778118) | 206374  (138674 to 303154) | 214816  (143055 to 316878) |
| Eastern Sub-Saharan Africa | 1436051  (1282887 to 1589453) | 3244072  (2869561 to 3609905) | 65472  (46025 to 93093) | 143294  (100041 to 205828) |
| High-income Asia Pacific | 2122794  (1903099 to 2348266) | 2769221  (2466596 to 3070249) | 83451  (56730 to 121337) | 113455  (79621 to 161746) |
| High-income North America | 3080307  (2766556 to 3406529) | 4279805  (3813432 to 4707427) | 118905  (80870 to 173610) | 165979  (114395 to 239719) |
| North Africa and Middle East | 6586898  (5939819 to 7292767) | 12976695  (11647714 to 14349490) | 276369  (189259 to 403521) | 539124  (370628 to 782669) |
| Oceania | 112742  (100089 to 126892) | 259189  (226992 to 291462) | 3656  (2353 to 5560) | 8294  (5272 to 12522) |
| South Asia | 32081349  (28516567 to 36037814) | 55485190  (49062021 to 62842997) | 1391910  (972604 to 1965521) | 2285352  (1576821 to 3281211) |
| Southeast Asia | 7178590  (6390500 to 8012839) | 11320903  (10179658 to 12496512) | 288187  (193142 to 422905) | 451649  (305473 to 661764) |
| Southern Latin America | 967604  (869171 to 1072960) | 1363645  (1215905 to 1512654) | 36046  (23944 to 53479) | 50358  (34087 to 74033) |
| Southern Sub-Saharan Africa | 602786  (538259 to 663849) | 1059805  (938589 to 1177798) | 25596  (17847 to 36935) | 44575  (31164 to 64303) |
| Tropical Latin America | 4067963  (3650308 to 4493560) | 6759436  (6077276 to 7435843) | 160632  (108646 to 237288) | 272422  (186998 to 398263) |
| Western Europe | 6730264  (6031222 to 7394682) | 8083424  (7223640 to 8953331) | 268013  (185734 to 388314) | 323694  (226166 to 466150) |
| Western Sub-Saharan Africa | 1717211  (1540538 to 1895977) | 4669907  (4157775 to 5186404) | 75876  (52404 to 108277) | 201501  (138395 to 290933) |

DALYs, disability adjusted life years; UI, uncertainty interval; SDI, Socio-demographic Index.
