# Supplementary material for: The burden of refraction disorders in 204 countries and territories from 1990 to 2021: A systematic analysis from the global burden of disease 2021
Source: Adv Ophthalmol Pract Res. 2024 Nov 6;5(2):79–87. doi: 10.1016/j.aopr.2024.11.001 (PMC11930593; doi:10.1016/j.aopr.2024.11.001)
Supplement: Multimedia component 3 [file mmc3.docx]

Supplementary Table 4. The burden of refraction disorders by gender in the global population in 2021.

| age | sex | Prevalence (95% UI) | ASPRs(95% UI) | DALY cases(95%UI) | Age-standardized DALY rate  per 100,000 people (95% UI) |
| --- | --- | --- | --- | --- | --- |
| <5 | Female | 1668875.45  (1329656.49 to 2049802.89) | 524.49  (417.88 to 644.2) | 56012.87  (34449.1 to 91623.42) | 17.6  (10.83 to 28.79) |
| <5 | Male | 1588516.42  (1247635.84 to 1947763.27) | 467.24  (366.97 to 572.91) | 53002.64  (32663.46 to 86135.55) | 15.59  (9.61 to 25.34) |
| 5-9 | Female | 3456358.83  (2585121.61 to 4453787.65) | 1039.27  (777.3 to 1339.17) | 121007.15  (74177.91 to 200012.31) | 36.38  (22.3 to 60.14) |
| 5-9 | Male | 3258867.86  (2414620.57 to 4223512.05) | 919.35  (681.18 to 1191.48) | 113089.91  (68467.73 to 184477.08) | 31.9  (19.32 to 52.04) |
| 10-14 | Female | 3846937.04  (2876341.14 to 4911878.77) | 1191.27  (890.71 to 1521.05) | 137858.23  (84582.41 to 210519.86) | 42.69  (26.19 to 65.19) |
| 10-14 | Male | 3578902.98  (2654305.48 to 4557681.28) | 1041.26  (772.25 to 1326.03) | 127997.38  (77535.01 to 196226.8) | 37.24  (22.56 to 57.09) |
| 15-19 | Female | 3669837.14  (2834792.75 to 4543259.32) | 1208.57  (933.57 to 1496.22) | 134814.96  (84533.58 to 205739.52) | 44.4  (27.84 to 67.76) |
| 15-19 | Male | 3385547.64  (2595897.89 to 4217988.69) | 1056.89  (810.38 to 1316.76) | 124275.24  (76824.85 to 190301.66) | 38.8  (23.98 to 59.41) |
| 20-24 | Female | 3504052.81  (2687799.35 to 4390460.06) | 1192.86  (914.99 to 1494.61) | 131116.57  (82745.75 to 202645.16) | 44.64  (28.17 to 68.99) |
| 20-24 | Male | 3203673.18  (2443292.18 to 4040025.48) | 1055.9  (805.29 to 1331.56) | 120104.39  (75095.33 to 187224.56) | 39.59  (24.75 to 61.71) |
| 25-29 | Female | 3470088.79  (2734288.9 to 4343861) | 1192.52  (939.66 to 1492.8) | 131012.68  (84482.78 to 198499.53) | 45.02  (29.03 to 68.22) |
| 25-29 | Male | 3190011.5  (2507152.67 to 4011977.26) | 1072.79  (843.15 to 1349.22) | 121135.61  (78069.8 to 184891.31) | 40.74  (26.25 to 62.18) |
| 30-34 | Female | 3637792.41  (2745162.74 to 4788186.25) | 1216.93  (918.33 to 1601.77) | 139968.83  (87875.66 to 212017.54) | 46.82  (29.4 to 70.93) |
| 30-34 | Male | 3377188.53  (2525246.99 to 4431170.79) | 1105.28  (826.46 to 1450.23) | 131209  (81701.55 to 198233.58) | 42.94  (26.74 to 64.88) |
| 35-39 | Female | 3943827.98  (3071958.01 to 4935343.44) | 1419.65  (1105.81 to 1776.56) | 153945.29  (97601.12 to 228898.88) | 55.42  (35.13 to 82.4) |
| 35-39 | Male | 3609869.97  (2795808.07 to 4533882.16) | 1275.29  (987.7 to 1601.72) | 143104.69  (90181.46 to 213952.52) | 50.56  (31.86 to 75.58) |
| 40-44 | Female | 4365637.78  (3226199.24 to 5600401.75) | 1759.7  (1300.42 to 2257.41) | 173875.76  (110630.43 to 264466.56) | 70.09  (44.59 to 106.6) |
| 40-44 | Male | 3931301.68  (2913804.52 to 5031908.6) | 1559.04  (1155.53 to 1995.51) | 159612.48  (101962.16 to 243004.94) | 63.3  (40.44 to 96.37) |
| 45-49 | Female | 5527930.15  (4303148.48 to 6911772.66) | 2345.9  (1826.13 to 2933.16) | 225434.38  (147808.56 to 339238.21) | 95.67  (62.73 to 143.96) |
| 45-49 | Male | 4901819.24  (3794744.4 to 6177644.75) | 2060.78  (1595.36 to 2597.15) | 204101.87  (132792.8 to 308321.51) | 85.81  (55.83 to 129.62) |
| 50-54 | Female | 6814605.47  (5172029.23 to 8699423.15) | 3056.66  (2319.89 to 3902.09) | 282701.28  (185164.6 to 428314.45) | 126.8  (83.05 to 192.12) |
| 50-54 | Male | 5989019  (4554014.57 to 7635802.95) | 2697.99  (2051.54 to 3439.85) | 253414.74  (165742.72 to 385780.28) | 114.16  (74.67 to 173.79) |
| 55-59 | Female | 8036879.5  (6181087.11 to 10377478.74) | 3998.38  (3075.11 to 5162.83) | 337824.37  (214258.93 to 513530.34) | 168.07  (106.59 to 255.48) |
| 55-59 | Male | 6983040.83  (5354063.59 to 8994931.28) | 3586.11  (2749.56 to 4619.31) | 297414.43  (188730.36 to 452750.14) | 152.74  (96.92 to 232.51) |
| 60-64 | Female | 8083817.19  (6197533.29 to 10571590.11) | 4913.85  (3767.25 to 6426.07) | 345567.95  (227210.51 to 517641.18) | 210.06  (138.11 to 314.65) |
| 60-64 | Male | 7010701.09  (5367849.57 to 9171755.71) | 4507.42  (3451.17 to 5896.84) | 301537.06  (196063.05 to 452263.29) | 193.87  (126.06 to 290.78) |
| 65-69 | Female | 8125730.95  (6404602.98 to 10252365.31) | 5642.52  (4447.37 to 7119.26) | 361362.85  (238893.87 to 534194.59) | 250.93  (165.89 to 370.95) |
| 65-69 | Male | 6982849.53  (5496524.21 to 8811874.66) | 5296.73  (4169.3 to 6684.11) | 308457.98  (204310.08 to 461900.78) | 233.98  (154.98 to 350.37) |
| 70-74 | Female | 6719174.58  (5264404.91 to 8628152.82) | 6139.16  (4809.97 to 7883.35) | 309660.89  (209029.61 to 452233.77) | 282.93  (190.99 to 413.2) |
| 70-74 | Male | 5662641.22  (4387416.3 to 7296497.83) | 5874.63  (4551.67 to 7569.66) | 256911.77  (171973.13 to 376490.57) | 266.53  (178.41 to 390.59) |
| 75-79 | Female | 4686484.11  (3707581.23 to 5744335.21) | 6500.19  (5142.44 to 7967.43) | 223436.24  (155512.75 to 317184.75) | 309.91  (215.7 to 439.94) |
| 75-79 | Male | 3802738.28  (3019861.43 to 4640599.54) | 6360.51  (5051.06 to 7761.92) | 177855.56  (123353.07 to 253013.48) | 297.48  (206.32 to 423.19) |
| 80-84 | Female | 3334317.91  (2635332.61 to 4240957.55) | 6546.71  (5174.3 to 8326.84) | 158865.11  (113020.55 to 226545.07) | 311.92  (221.91 to 444.81) |
| 80-84 | Male | 2325877.57  (1836754.33 to 2952803.59) | 6345.86  (5011.35 to 8056.35) | 109241.94  (77801.45 to 155500.34) | 298.05  (212.27 to 424.26) |
| 85-89 | Female | 1783794.68  (1392979.45 to 2272641.96) | 6265.72  (4892.95 to 7982.83) | 84491.43  (58751.5 to 116633.08) | 296.78  (206.37 to 409.68) |
| 85-89 | Male | 1056501.13  (832501.11 to 1342074.59) | 6123.7  (4825.35 to 7778.94) | 49271.34  (34142.5 to 68482.8) | 285.59  (197.9 to 396.94) |
| 90-94 | Female | 670186.8  (494559.59 to 862636.71) | 5556.7  (4100.53 to 7152.36) | 31273.64  (20958.44 to 45295.1) | 259.3  (173.77 to 375.55) |
| 90-94 | Male | 313707.39  (231894.27 to 404060.91) | 5382.3  (3978.63 to 6932.5) | 14332.29  (9602.65 to 20701.14) | 245.9  (164.75 to 355.17) |
| 95+ | Female | 196005.5  (141736.69 to 262518.18) | 4976.95  (3598.96 to 6665.83) | 9092.6  (6054.16 to 13192.82) | 230.88  (153.73 to 334.99) |
| 95+ | Male | 70806.56  (51746.46 to 94694.17) | 4682.84  (3422.29 to 6262.67) | 3206.66  (2100.37 to 4643.33) | 212.07  (138.91 to 307.09) |

DALYs, disability adjusted life years; UI, uncertainty interval.
