# Supplementary material for: The burden of refraction disorders in 204 countries and territories from 1990 to 2021: A systematic analysis from the global burden of disease 2021
Source: Adv Ophthalmol Pract Res. 2024 Nov 6;5(2):79–87. doi: 10.1016/j.aopr.2024.11.001 (PMC11930593; doi:10.1016/j.aopr.2024.11.001)

Supplementary Figure 1. Refraction disorder related burden with different geographic regions.
Age-standardized Prevalence(A) and DALY (B) rates of refraction disorders.
DALY, disability-adjusted life year; SDI, Socio-demographic Index.

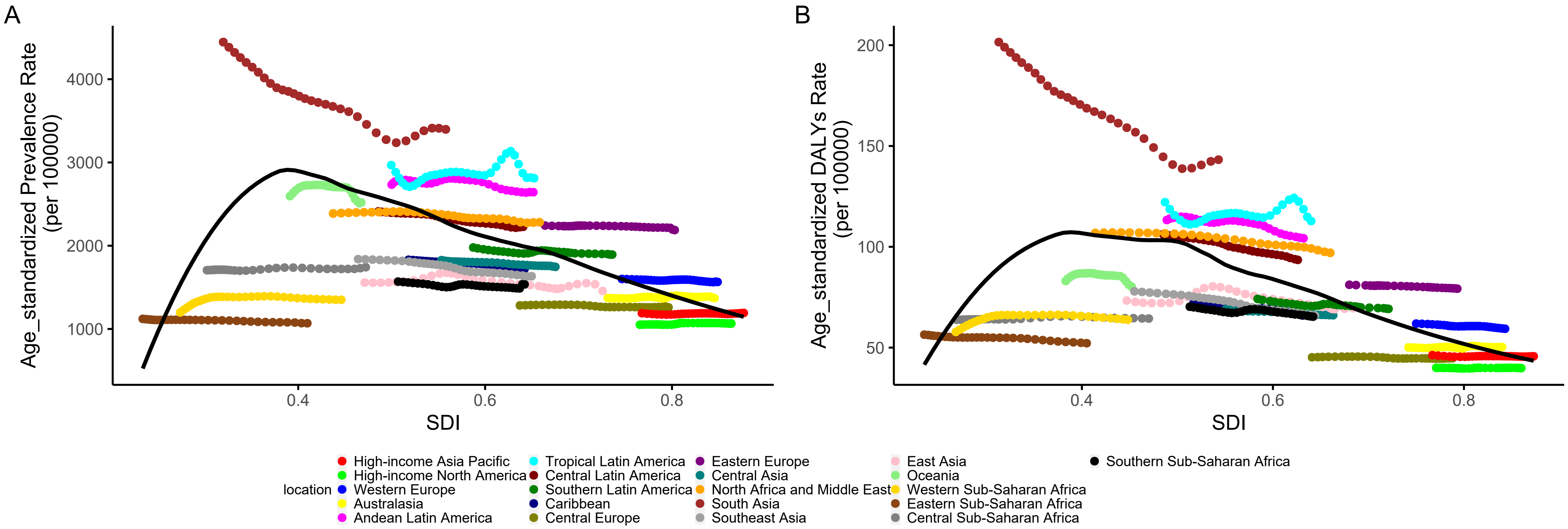

Supplement: Multimedia component 4 [file mmc4.pdf]
