# Supplementary material for: The burden of refraction disorders in 204 countries and territories from 1990 to 2021: A systematic analysis from the global burden of disease 2021
Source: Adv Ophthalmol Pract Res. 2024 Nov 6;5(2):79–87. doi: 10.1016/j.aopr.2024.11.001 (PMC11930593; doi:10.1016/j.aopr.2024.11.001)

Supplementary Figure 2.The burden of refraction disorder in different sexes and age groups in 2021 and change in different genders in 1990 and 2021.
Prevalence(A) and DALY (B) rates of refraction disorder.
Prevalence(C) and DALY (D) numbers of refraction disorder.
DALY, disability-adjusted life year;

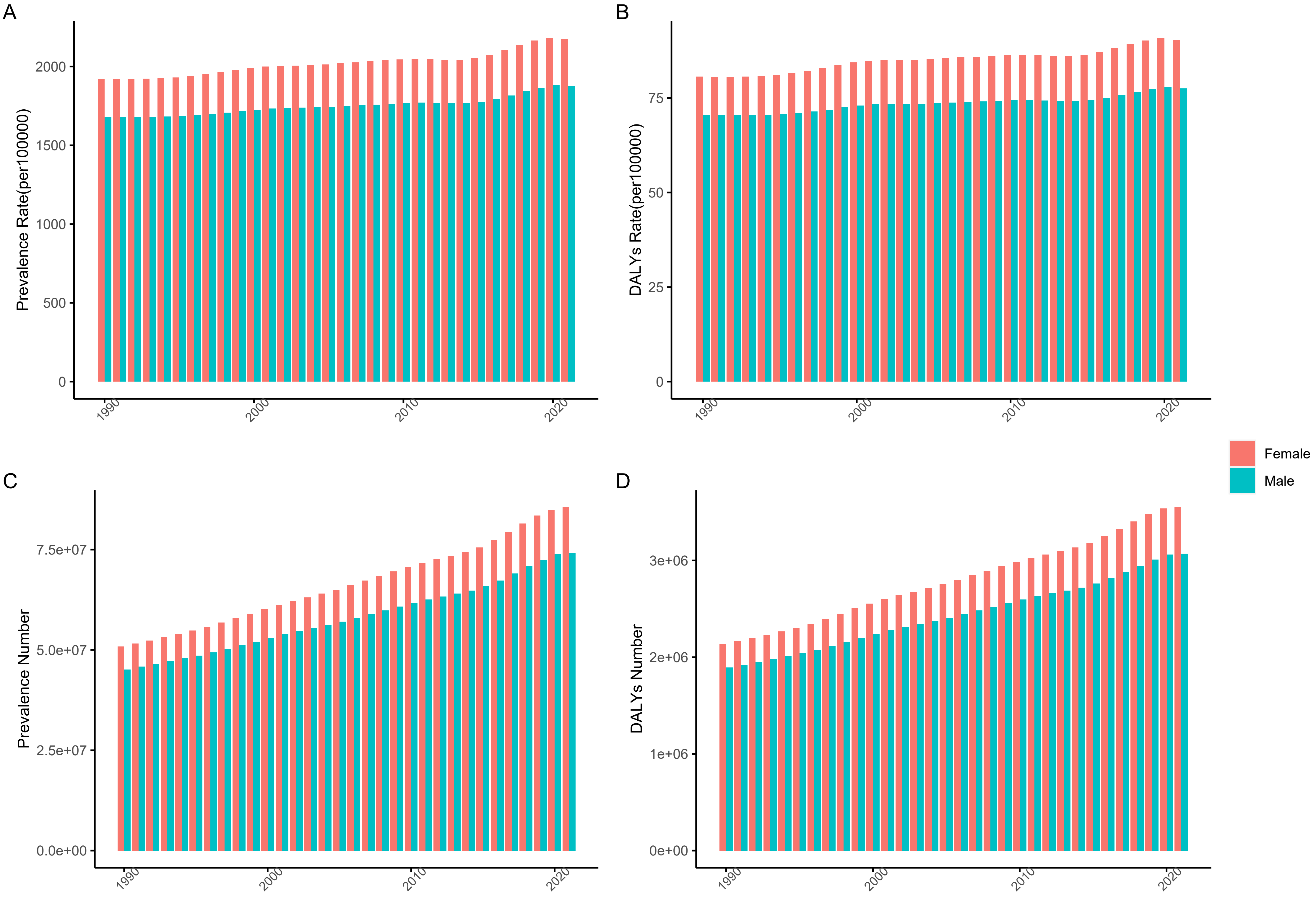

Supplement: Multimedia component 5 [file mmc5.pdf]
