# Supplementary material for: The burden of refraction disorders in 204 countries and territories from 1990 to 2021: A systematic analysis from the global burden of disease 2021
Source: Adv Ophthalmol Pract Res. 2024 Nov 6;5(2):79–87. doi: 10.1016/j.aopr.2024.11.001 (PMC11930593; doi:10.1016/j.aopr.2024.11.001)
Supplement: Multimedia component 6 [file mmc6.docx]

Supplementary Table 3. Age-standardized rates of prevalence and DALY due to Refraction disorders in 204 countries and regions in 2021, and their average annual percent changes from 1990 to 2021.

| location | Prevalence | | | | DALYs | | | |
| --- | --- | --- | --- | --- | --- | --- | --- | --- |
|  | Cases(95%UI) | Age-standardized Prevalence rate  per 100,000 people (95%UI) | AAPC,1990-2021  (95%CI) | P Value | Cases(95%UI) | Age-standardized DALY rate  per 100,000 people (95%UI) | AAPC,1990-2021  (95%CI) | P Value |
| Afghanistan | 600830.48  (527084.13 to 697092.82) | 2575.88 (2287.94 to 2874.74) | -0.18 (-0.22 to -0.15) | <0.001 | 23858.92  (16054.73 to 35308.43) | 109.77 (76.42 to 156.44) | -0.3 (-0.32 to -0.27) | <0.001 |
| Albania | 43419.2  (37709.55 to 49673.53) | 1254 (1107.48 to 1402.06) | -0.03 (-0.04 to -0.01) | <0.001 | 1556.21  (1032.06 to 2300.76) | 44.75 (29.67 to 66.32) | -0.02 (-0.05 to 0.02) | 0.402 |
| Algeria | 961070.2  (862500.95 to 1077983.81) | 2331.36 (2089.62 to 2603.2) | -0.31 (-0.32 to -0.3) | <0.001 | 38912.77  (26536.55 to 57031.51) | 95.71 (66.12 to 139.34) | -0.47 (-0.48 to -0.46) | <0.001 |
| American Samoa | 1076.52  (926.99 to 1239.78) | 2145.97 (1851.64 to 2460.19) | -0.19 (-0.22 to -0.16) | <0.001 | 34.33  (21.79 to 52.57) | 68.08 (43.12 to 104.32) | -0.26 (-0.28 to -0.24) | <0.001 |
| Andorra | 1360.71  (1210.33 to 1510.55) | 1406.55 (1253.88 to 1559.88) | -0.05 (-0.06 to -0.04) | <0.001 | 53.34  (37.08 to 76.55) | 52.53 (35.09 to 77.59) | -0.1 (-0.11 to -0.09) | <0.001 |
| Angola | 426278.4  (379844.58 to 476141.46) | 2521.12 (2233.6 to 2818.51) | -0.11 (-0.13 to -0.08) | <0.001 | 15401.9  (10265.43 to 22788.59) | 94.29 (64.38 to 136.34) | -0.2 (-0.21 to -0.18) | <0.001 |
| Antigua and Barbuda | 1388.31  (1228.83 to 1570.03) | 1420.9 (1258.19 to 1593.21) | -0.2 (-0.22 to -0.19) | <0.001 | 52.65  (35.3 to 78.25) | 53.26 (36.1 to 78.74) | -0.32 (-0.36 to -0.27) | <0.001 |
| Argentina | 951585.96  (849513.57 to 1054095.12) | 1973.22 (1763.09 to 2191.23) | -0.12 (-0.13 to -0.11) | <0.001 | 35964.2  (24405.77 to 52806.69) | 73.43 (49.01 to 107.95) | -0.22 (-0.24 to -0.21) | <0.001 |
| Armenia | 63902.21  (55562.67 to 72981.16) | 1784.46 (1566.73 to 2005.45) | -0.15 (-0.16 to -0.14) | <0.001 | 2429.76  (1621.58 to 3577.36) | 67.03 (44.82 to 99.59) | -0.2 (-0.22 to -0.17) | <0.001 |
| Australia | 379677.44  (338055.15 to 418641.75) | 1358 (1210.64 to 1508.06) | 0.01 (-0.05 to 0.06) | 0.778 | 14301.1  (9806.57 to 20790.08) | 49.23 (32.63 to 73.03) | -0.01 (-0.06 to 0.04) | 0.684 |
| Austria | 149027.07  (132983.2 to 164525.95) | 1409.98 (1261.74 to 1559.53) | -0.09 (-0.1 to -0.07) | <0.001 | 5905.1  (4119.36 to 8431.99) | 52.91 (36.09 to 77.35) | -0.14 (-0.17 to -0.12) | <0.001 |
| Azerbaijan | 194303.98  (170788.61 to 222187.28) | 1791.14 (1583.69 to 2023.86) | -0.12 (-0.13 to -0.11) | <0.001 | 7324.03  (4861.94 to 10780.84) | 67.63 (45.6 to 99.26) | -0.15 (-0.17 to -0.13) | <0.001 |
| Bahamas | 5801.74  (5141.82 to 6555.46) | 1416.51 (1258.39 to 1587.69) | -0.17 (-0.18 to -0.16) | <0.001 | 218.39  (146.01 to 323.16) | 53.13 (35.61 to 78.66) | -0.27 (-0.3 to -0.23) | <0.001 |
| Bahrain | 31353.99  (27843.31 to 34915.99) | 2297.61 (2040.54 to 2541.49) | -0.35 (-0.36 to -0.34) | <0.001 | 1249.18  (849.14 to 1863.78) | 93.53 (64.86 to 134.9) | -0.52 (-0.54 to -0.5) | <0.001 |
| Bangladesh | 4466276.19  (3922484.1 to 5051647.91) | 2977.82 (2601.48 to 3367.82) | -0.22 (-0.26 to -0.19) | <0.001 | 171942.81  (114448.09 to 253224.03) | 115.32 (76.86 to 168.07) | -0.46 (-0.49 to -0.44) | <0.001 |
| Barbados | 2654.71  (2351.4 to 2996.64) | 748.8 (665.92 to 830.7) | -0.12 (-0.13 to -0.12) | <0.001 | 103.9  (69.97 to 151.62) | 28.3 (19.09 to 41.64) | -0.2 (-0.23 to -0.18) | <0.001 |
| Belarus | 230834.88  (201256.83 to 264743.09) | 1832.5 (1619.11 to 2065.13) | -0.11 (-0.11 to -0.1) | <0.001 | 8009.45  (5229.65 to 11978.05) | 63.75 (41.96 to 95.61) | -0.15 (-0.17 to -0.13) | <0.001 |
| Belgium | 190415.9  (170688.56 to 211858.39) | 1413.66 (1268.13 to 1565.96) | -0.1 (-0.12 to -0.08) | <0.001 | 7510.15  (5243.64 to 10734.25) | 52.93 (36.03 to 78.39) | -0.18 (-0.2 to -0.17) | <0.001 |
| Belize | 5853.54  (5206.81 to 6559.39) | 1449.98 (1292.97 to 1621.69) | -0.21 (-0.21 to -0.2) | <0.001 | 218.21  (144.85 to 327.82) | 55.23 (37.19 to 81.95) | -0.32 (-0.34 to -0.31) | <0.001 |
| Benin | 124220.9  (109902.33 to 137977.47) | 1451.25 (1272.78 to 1636.78) | 1.56 (1.5 to 1.62) | <0.001 | 5584.34  (3841.77 to 7956.53) | 71.26 (50.32 to 98.14) | 1.45 (1.41 to 1.5) | <0.001 |
| Bermuda | 1208.32  (1062.74 to 1363.83) | 1506.12 (1327.38 to 1680.33) | -0.16 (-0.18 to -0.15) | <0.001 | 47.62  (32.44 to 69.73) | 56.94 (38.23 to 84.27) | -0.26 (-0.28 to -0.23) | <0.001 |
| Bhutan | 14409.8  (12865.63 to 16174.98) | 2202.02 (1969.94 to 2485.38) | -0.52 (-0.55 to -0.49) | <0.001 | 627  (448.89 to 887.74) | 97.06 (69.65 to 136.39) | -0.74 (-0.79 to -0.68) | <0.001 |
| Bolivia (Plurinational State of) | 262066.57  (235375.84 to 291157.82) | 2342.84 (2105.07 to 2612.89) | -0.17 (-0.18 to -0.16) | <0.001 | 10242.05  (6880.03 to 15140.79) | 93.14 (63.21 to 137.28) | -0.3 (-0.32 to -0.28) | <0.001 |
| Bosnia and Herzegovina | 57999.19  (50804.43 to 66366.9) | 1247.52 (1106.6 to 1403.05) | -0.08 (-0.09 to -0.07) | <0.001 | 2053.59  (1353.19 to 3013.22) | 44.04 (29.44 to 65.21) | -0.1 (-0.12 to -0.08) | <0.001 |
| Botswana | 29204.21  (25612.88 to 32677.13) | 1473.05 (1295.61 to 1663.33) | -0.26 (-0.28 to -0.25) | <0.001 | 1173.43  (795.69 to 1729.48) | 60.44 (41.71 to 87.36) | -0.51 (-0.52 to -0.49) | <0.001 |
| Brazil | 6526679.49  (5865407.8 to 7187414.72) | 2795.57 (2508.72 to 3071.01) | -0.16 (-0.28 to -0.05) | 0.006 | 264118.35  (181519.86 to 386091.05) | 111.97 (76.91 to 162.77) | -0.26 (-0.36 to -0.16) | <0.001 |
| Brunei Darussalam | 5679.62  (5030.08 to 6335.32) | 1308.81 (1168.89 to 1450.38) | -0.06 (-0.08 to -0.04) | <0.001 | 212.31  (141.53 to 315.33) | 49.59 (33.47 to 73.29) | -0.11 (-0.14 to -0.07) | <0.001 |
| Bulgaria | 140439.16  (122134.3 to 161347.64) | 1336.96 (1187.53 to 1498.38) | -0.07 (-0.09 to -0.05) | <0.001 | 5015.31  (3417.97 to 7349.44) | 47.42 (31.92 to 70.37) | -0.07 (-0.11 to -0.03) | <0.001 |
| Burkina Faso | 205140.43  (180169.93 to 229564.81) | 1408.06 (1232.59 to 1599.8) | 1.64 (1.53 to 1.75) | <0.001 | 8861.79  (6063.52 to 12980.06) | 64.99 (45.44 to 91.65) | 1.58 (1.49 to 1.68) | <0.001 |
| Burundi | 66855.25  (58967.92 to 74728.39) | 805.99 (711.57 to 897.96) | -0.12 (-0.16 to -0.09) | <0.001 | 2822.87  (1966 to 4099.25) | 36.96 (26.22 to 51.79) | -0.21 (-0.25 to -0.17) | <0.001 |
| Cabo Verde | 5308.22  (4712.65 to 5977.73) | 1031.86 (910.07 to 1169.43) | -0.31 (-0.32 to -0.29) | <0.001 | 243.86  (168.63 to 347.56) | 48.51 (34.01 to 68.53) | -0.53 (-0.56 to -0.5) | <0.001 |
| Cambodia | 383535.08  (342784.92 to 429144.51) | 2493.83 (2219.72 to 2787.66) | -0.5 (-0.61 to -0.4) | <0.001 | 15194.19  (10198.55 to 21867.2) | 101.22 (68.61 to 144.7) | -0.71 (-0.78 to -0.64) | <0.001 |
| Cameroon | 215554.43  (190473.13 to 239966.13) | 963.32 (853.87 to 1082.79) | -0.23 (-0.4 to -0.06) | 0.008 | 9164.44  (6199.95 to 13266.17) | 45.2 (31.81 to 63.42) | -0.33 (-0.45 to -0.22) | <0.001 |
| Canada | 411219.49  (368226.2 to 455459.76) | 991.1 (886.11 to 1099.73) | -0.03 (-0.05 to -0.01) | 0.001 | 16345.61  (11442.12 to 23483.46) | 37.41 (25.1 to 55.26) | -0.07 (-0.09 to -0.06) | <0.001 |
| Central African Republic | 51673.2  (45434.36 to 57205.8) | 1520.78 (1346.82 to 1701.29) | 0.22 (0.19 to 0.26) | <0.001 | 1728.94  (1124.6 to 2639.51) | 52.21 (34.8 to 77.08) | 0.22 (0.19 to 0.25) | <0.001 |
| Chad | 135627.43  (120807.64 to 151295.74) | 1255.95 (1114.54 to 1410.81) | 0.71 (0.66 to 0.76) | <0.001 | 5811.39  (3928.98 to 8151.1) | 60.04 (42.27 to 83.95) | 0.57 (0.52 to 0.62) | <0.001 |
| Chile | 345368.8  (309380.97 to 385065.33) | 1745.93 (1560.92 to 1941.49) | -0.12 (-0.14 to -0.1) | <0.001 | 11939.38  (7849.93 to 17858.9) | 59.61 (38.43 to 89.61) | -0.17 (-0.2 to -0.15) | <0.001 |
| China | 26970569.17  (23579348.17 to 30840249.04) | 1467.89 (1301.18 to 1641.36) | -0.18 (-0.22 to -0.14) | <0.001 | 1238861.61  (876580.05 to 1720626.54) | 65.89 (46.69 to 92.2) | -0.34 (-0.41 to -0.28) | <0.001 |
| Colombia | 1198738.35  (1068595.37 to 1339974.15) | 2285.43 (2044.21 to 2542.51) | -0.24 (-0.26 to -0.22) | <0.001 | 49046.97  (33717.81 to 71208.35) | 92.88 (63.72 to 135.28) | -0.38 (-0.41 to -0.35) | <0.001 |
| Comoros | 7271.19  (6433.94 to 8218.85) | 1189.98 (1042.5 to 1348.81) | -0.44 (-0.46 to -0.42) | <0.001 | 334.02  (236.45 to 477.74) | 56.72 (40.62 to 80.02) | -0.63 (-0.64 to -0.62) | <0.001 |
| Congo | 98510.04  (87211.11 to 110732.78) | 2846.83 (2501.34 to 3210.85) | -0.16 (-0.19 to -0.14) | <0.001 | 3499.89  (2307.1 to 5194.04) | 103.45 (69.67 to 151.53) | -0.2 (-0.23 to -0.18) | <0.001 |
| Cook Islands | 498.44  (428.64 to 580.28) | 2131.81 (1853.95 to 2439.93) | -0.21 (-0.23 to -0.2) | <0.001 | 15.78  (9.83 to 24.03) | 68.04 (43.33 to 102.56) | -0.31 (-0.33 to -0.29) | <0.001 |
| Costa Rica | 96634.9  (86292.43 to 108213.99) | 1890.87 (1694.6 to 2099.94) | -0.22 (-0.26 to -0.17) | <0.001 | 3884.6  (2638.42 to 5700.3) | 75.38 (51.25 to 111.08) | -0.35 (-0.4 to -0.3) | <0.001 |
| Côte d'Ivoire | 263871.28  (233173.28 to 295308.34) | 1438.28 (1268.78 to 1638.83) | 2.28 (2.18 to 2.38) | <0.001 | 11484.58  (7875.69 to 16689.89) | 67.69 (47.47 to 95.39) | 2.36 (2.3 to 2.43) | <0.001 |
| Croatia | 77978.31  (67151.41 to 89465.42) | 1245.04 (1098.12 to 1405.91) | -0.06 (-0.07 to -0.05) | <0.001 | 2760.46  (1840.38 to 4019.28) | 43.9 (28.74 to 64.22) | -0.06 (-0.08 to -0.04) | <0.001 |
| Cuba | 257986.61  (228129.44 to 292679) | 1934.4 (1726.91 to 2173.11) | -0.14 (-0.14 to -0.13) | <0.001 | 10203.15  (6910.31 to 14968.56) | 73.94 (49.54 to 109.59) | -0.22 (-0.24 to -0.2) | <0.001 |
| Cyprus | 20742.47  (18497.15 to 23122.1) | 1406.99 (1249.87 to 1562.04) | -0.1 (-0.11 to -0.09) | <0.001 | 802.85  (548.77 to 1170.99) | 52.71 (36.03 to 77.88) | -0.17 (-0.18 to -0.16) | <0.001 |
| Czechia | 189105.73  (163531.35 to 216044.06) | 1231.65 (1085.95 to 1384.93) | -0.08 (-0.11 to -0.06) | <0.001 | 6661.18  (4463.02 to 9828.86) | 43.27 (28.15 to 64.31) | -0.06 (-0.1 to -0.03) | <0.001 |
| Democratic People's Republic of Korea | 356332.19  (311190.09 to 403836.86) | 1183.8 (1039.67 to 1340.94) | -0.43 (-0.47 to -0.4) | <0.001 | 13077.48  (8730.58 to 19003.27) | 43.19 (28.9 to 63.85) | -0.71 (-0.74 to -0.68) | <0.001 |
| Democratic Republic of the Congo | 764916.83  (678863.9 to 853349.01) | 1391.85 (1232.89 to 1558.79) | 0.07 (0.05 to 0.08) | <0.001 | 27426.31  (18096.18 to 41066.21) | 52.41 (35.57 to 76.29) | 0.11 (0.08 to 0.14) | <0.001 |
| Denmark | 102463.74  (91389.58 to 113444.8) | 1505.83 (1341.08 to 1668.25) | -0.07 (-0.08 to -0.06) | <0.001 | 4045.23  (2783.99 to 5867.38) | 56.23 (37.51 to 83.16) | -0.14 (-0.17 to -0.1) | <0.001 |
| Djibouti | 11518.77  (10086.67 to 12977.46) | 1210.2 (1063.97 to 1362.81) | -0.43 (-0.44 to -0.42) | <0.001 | 518.22  (359.02 to 735.5) | 57.83 (41.14 to 81.02) | -0.63 (-0.64 to -0.61) | <0.001 |
| Dominica | 1042.03  (924.16 to 1173.58) | 1418.96 (1263.75 to 1592.24) | -0.22 (-0.23 to -0.2) | <0.001 | 39.78  (26.76 to 59.04) | 53.48 (35.92 to 80) | -0.33 (-0.35 to -0.32) | <0.001 |
| Dominican Republic | 226687.2  (202017.97 to 253953.55) | 2083.47 (1859.13 to 2325.88) | -0.22 (-0.23 to -0.21) | <0.001 | 8852.59  (6042.92 to 13045.06) | 81.94 (56.15 to 120.51) | -0.34 (-0.36 to -0.33) | <0.001 |
| Ecuador | 303262.31  (271364.86 to 337667.99) | 1696.2 (1522.47 to 1886.55) | -0.11 (-0.12 to -0.1) | <0.001 | 12001.07  (8183.7 to 17488.49) | 67.74 (46.61 to 98.09) | -0.19 (-0.21 to -0.18) | <0.001 |
| Egypt | 2285490.51  (2035745.26 to 2533977.57) | 2513.09 (2232.37 to 2797.9) | 0.12 (0.11 to 0.14) | <0.001 | 91711.47  (61919.43 to 134177.41) | 104.36 (72.62 to 149.36) | 0.05 (0.03 to 0.08) | <0.001 |
| El Salvador | 166044.66  (149549.8 to 183152.93) | 2598.6 (2336.5 to 2868.46) | -0.26 (-0.28 to -0.25) | <0.001 | 6674.17  (4643.03 to 9753.32) | 104.84 (72.84 to 153.65) | -0.44 (-0.46 to -0.42) | <0.001 |
| Equatorial Guinea | 18502.98  (16498.03 to 20527.22) | 2328.15 (2068.34 to 2634.67) | -0.19 (-0.23 to -0.14) | <0.001 | 644.35  (425.55 to 963.71) | 82.84 (56.14 to 121.13) | -0.3 (-0.35 to -0.25) | <0.001 |
| Eritrea | 62297.96  (54826.87 to 70732.18) | 1401.71 (1240.56 to 1596.89) | -0.37 (-0.42 to -0.32) | <0.001 | 3162.02  (2268.54 to 4347.05) | 76.75 (56.02 to 104.26) | -0.64 (-0.7 to -0.59) | <0.001 |
| Estonia | 22874.68  (19902.02 to 26247.54) | 1204.02 (1059.38 to 1359.06) | -0.11 (-0.12 to -0.1) | <0.001 | 800.02  (540.87 to 1199.94) | 42.27 (28.12 to 63.51) | -0.14 (-0.17 to -0.12) | <0.001 |
| Eswatini | 13625.32  (12190.1 to 15116.98) | 1614.64 (1435.42 to 1808.43) | -0.22 (-0.24 to -0.21) | <0.001 | 551.88  (379.12 to 805.68) | 68.72 (48.29 to 98.61) | -0.42 (-0.45 to -0.39) | <0.001 |
| Ethiopia | 907324.73  (800630.51 to 1018040.2) | 1015.87 (904.09 to 1136.69) | 0.27 (0.21 to 0.33) | <0.001 | 38051  (25494.83 to 54404.54) | 45.12 (31.6 to 63.53) | 0.23 (0.21 to 0.26) | <0.001 |
| Fiji | 19786.04  (17121.69 to 22755.43) | 2351.2 (2042.8 to 2690.77) | -0.23 (-0.26 to -0.21) | <0.001 | 637.72  (397.09 to 966.07) | 75.03 (46.62 to 113.46) | -0.3 (-0.38 to -0.21) | <0.001 |
| Finland | 93591.37  (83013.09 to 104592.3) | 1402.68 (1250.89 to 1545.39) | -0.07 (-0.08 to -0.07) | <0.001 | 3647.99  (2548.82 to 5229.92) | 51.75 (34.85 to 76.32) | -0.15 (-0.16 to -0.13) | <0.001 |
| France | 903894.15  (799364.85 to 1007157.1) | 1123.46 (996.99 to 1250.81) | -0.02 (-0.08 to 0.05) | 0.616 | 37442.4  (26002.65 to 53432.52) | 44.06 (29.94 to 63.95) | -0.11 (-0.26 to 0.04) | 0.135 |
| Gabon | 31803.91  (28132.44 to 35994.05) | 2550.51 (2247.14 to 2880.66) | -0.16 (-0.17 to -0.14) | <0.001 | 1147.41  (763.61 to 1686.46) | 93.41 (63.18 to 134.7) | -0.24 (-0.26 to -0.22) | <0.001 |
| Gambia | 23408.99  (20465.92 to 26374.51) | 1501.27 (1304.91 to 1721.7) | 0.04 (0 to 0.08) | 0.029 | 950.87  (643.27 to 1406.65) | 63.85 (44.16 to 91.61) | -0.03 (-0.08 to 0.01) | 0.191 |
| Georgia | 82059.69  (71357.33 to 94809.1) | 1802.65 (1592.43 to 2031.15) | -0.1 (-0.11 to -0.09) | <0.001 | 3125.7  (2124.3 to 4551.99) | 67.75 (46.21 to 99.95) | -0.12 (-0.15 to -0.1) | <0.001 |
| Germany | 1442873.41  (1283324.32 to 1606863.03) | 1391.51 (1233.92 to 1544.3) | -0.07 (-0.08 to -0.06) | <0.001 | 57190.82  (40175.25 to 81647.72) | 51.84 (34.81 to 77.18) | -0.13 (-0.14 to -0.11) | <0.001 |
| Ghana | 441664.24  (393000.62 to 492669.96) | 1778.62 (1565.7 to 2009.55) | 0.82 (0.77 to 0.88) | <0.001 | 18024.87  (12275.92 to 26286.75) | 76.91 (53.48 to 110.55) | 0.81 (0.75 to 0.87) | <0.001 |
| Greece | 183060.46  (161931.3 to 204743.27) | 1448.77 (1285.37 to 1622.04) | -0.14 (-0.15 to -0.13) | <0.001 | 7413.03  (5239.34 to 10547.24) | 54.45 (37 to 80.39) | -0.27 (-0.29 to -0.26) | <0.001 |
| Greenland | 558.44  (495.5 to 620.23) | 994.6 (886.34 to 1103.23) | -0.05 (-0.07 to -0.04) | <0.001 | 21.49  (14.42 to 31.8) | 37.65 (25.1 to 55.72) | -0.1 (-0.11 to -0.08) | <0.001 |
| Grenada | 1555.75  (1383.42 to 1749.05) | 1431.5 (1281.2 to 1597.5) | -0.24 (-0.25 to -0.23) | <0.001 | 59.15  (40.45 to 87.11) | 54.09 (36.86 to 80.1) | -0.36 (-0.38 to -0.34) | <0.001 |
| Guam | 4184.79  (3572.25 to 4885.19) | 2153.2 (1842.75 to 2479.86) | -0.18 (-0.26 to -0.1) | <0.001 | 133.83  (83.86 to 204.61) | 69.23 (43.9 to 105.83) | -0.22 (-0.31 to -0.13) | <0.001 |
| Guatemala | 361496.93  (322364.28 to 400866.49) | 2474.31 (2211.26 to 2740.77) | -0.26 (-0.28 to -0.24) | <0.001 | 14348.29  (9774.07 to 21037.41) | 101.62 (70.09 to 146.55) | -0.43 (-0.45 to -0.4) | <0.001 |
| Guinea | 126952.72  (112150.68 to 142568.35) | 1446.61 (1270.64 to 1653.32) | 0.63 (0.61 to 0.65) | <0.001 | 5618.12  (3911.81 to 8046.91) | 69.24 (48.86 to 96.53) | 0.51 (0.47 to 0.55) | <0.001 |
| Guinea-Bissau | 19824.58  (17528.38 to 22126.13) | 1588.43 (1396.04 to 1789.25) | 0.36 (0.31 to 0.4) | <0.001 | 875.97  (606.59 to 1258.99) | 77.52 (55.17 to 107.13) | 0.25 (0.16 to 0.34) | <0.001 |
| Guyana | 10614.82  (9420.09 to 11968.25) | 1433.37 (1271.53 to 1606.36) | -0.22 (-0.23 to -0.21) | <0.001 | 395.84  (265.15 to 583.99) | 54.03 (36.45 to 78.94) | -0.35 (-0.38 to -0.31) | <0.001 |
| Haiti | 166959.66  (148040.97 to 186503.46) | 1466.58 (1305.15 to 1629.59) | -0.18 (-0.19 to -0.17) | <0.001 | 6257.23  (4122.56 to 9382.47) | 56.87 (38.52 to 84.19) | -0.3 (-0.32 to -0.27) | <0.001 |
| Honduras | 198327.62  (177387.12 to 223486.15) | 2227.63 (1980.93 to 2474.14) | -0.23 (-0.26 to -0.2) | <0.001 | 7549.42  (5110.39 to 11153.85) | 87.77 (60.69 to 128.15) | -0.38 (-0.41 to -0.35) | <0.001 |
| Hungary | 150334.01  (130778.17 to 171037.2) | 1122.71 (990.13 to 1259.53) | -0.07 (-0.1 to -0.04) | <0.001 | 5304.67  (3524.96 to 7769.84) | 39.47 (26.15 to 59.69) | -0.05 (-0.09 to -0.01) | 0.02 |
| Iceland | 5036.49  (4426.04 to 5631.89) | 1308.03 (1147.74 to 1448.06) | -0.07 (-0.08 to -0.06) | <0.001 | 197.99  (134.93 to 284.34) | 49.67 (33.19 to 72.76) | -0.13 (-0.15 to -0.1) | <0.001 |
| India | 45201315.23  (39968921.35 to 51317627.73) | 3495.7 (3079.16 to 3974.25) | -1.01 (-1.05 to -0.96) | <0.001 | 1880365.45  (1300989.82 to 2683835.26) | 146.97 (102.34 to 207.98) | -1.3 (-1.38 to -1.22) | <0.001 |
| Indonesia | 3572905.46  (3164180.29 to 3984665.9) | 1311.56 (1169.7 to 1460.37) | -0.48 (-0.52 to -0.45) | <0.001 | 146292.67  (99769.48 to 214650.54) | 54.11 (37.12 to 79.06) | -0.64 (-0.67 to -0.62) | <0.001 |
| Iran (Islamic Republic of) | 2276622.66  (2044745.24 to 2514059.15) | 2743.7 (2470.71 to 3023.1) | -0.37 (-0.41 to -0.32) | <0.001 | 107783.87  (77136.16 to 150891.84) | 130.81 (93.78 to 182.09) | -0.55 (-0.64 to -0.46) | <0.001 |
| Iraq | 807560.26  (717592.14 to 902562.05) | 2289.45 (2038.07 to 2569.22) | -0.32 (-0.34 to -0.31) | <0.001 | 31728.53  (21619.04 to 47090.84) | 93.41 (65.41 to 133.7) | -0.51 (-0.53 to -0.5) | <0.001 |
| Ireland | 76024.35  (67571.1 to 84077.56) | 1405.01 (1258.03 to 1552.48) | -0.11 (-0.12 to -0.1) | <0.001 | 2955.43  (2056.44 to 4286.05) | 52.67 (36.17 to 76.81) | -0.18 (-0.21 to -0.16) | <0.001 |
| Israel | 142262.21  (126809.04 to 157281.94) | 1421.49 (1263.06 to 1577.54) | -0.06 (-0.07 to -0.06) | <0.001 | 5463.03  (3734.53 to 8116.53) | 53.67 (36.34 to 80.42) | -0.13 (-0.14 to -0.12) | <0.001 |
| Italy | 1287802.68  (1152541.4 to 1425479.77) | 1876.33 (1688.12 to 2077.68) | -0.07 (-0.09 to -0.06) | <0.001 | 51635.95  (36081.15 to 75078.76) | 70.56 (46.84 to 104.33) | -0.16 (-0.17 to -0.15) | <0.001 |
| Jamaica | 42261.03  (37499.64 to 47141.48) | 1424.67 (1263.87 to 1587.93) | -0.21 (-0.23 to -0.19) | <0.001 | 1607.9  (1074.76 to 2376.61) | 53.92 (35.98 to 79.57) | -0.31 (-0.34 to -0.28) | <0.001 |
| Japan | 1875770.17  (1670981.26 to 2090860.34) | 1109.45 (992.32 to 1226.36) | -0.02 (-0.04 to -0.01) | <0.001 | 78208.08  (54942.33 to 111588.87) | 42.97 (29.12 to 62.89) | -0.05 (-0.06 to -0.03) | <0.001 |
| Jordan | 228298.47  (201803.68 to 254462.03) | 2220.25 (1965.28 to 2464.51) | -0.38 (-0.46 to -0.31) | <0.001 | 9346.37  (6407.41 to 13563.54) | 95.15 (66.39 to 135.42) | -0.55 (-0.62 to -0.49) | <0.001 |
| Kazakhstan | 338243.04  (298477.02 to 382276.04) | 1792.44 (1587.35 to 2014.21) | -0.12 (-0.14 to -0.1) | <0.001 | 12628.12  (8472.43 to 18685.91) | 67.08 (45.38 to 98.93) | -0.14 (-0.17 to -0.12) | <0.001 |
| Kenya | 366472.33  (323083.82 to 409973.63) | 940.87 (834.23 to 1059.78) | -0.14 (-0.17 to -0.11) | <0.001 | 17166.24  (12032.68 to 24423.52) | 47.3 (34.13 to 65.85) | -0.45 (-0.48 to -0.43) | <0.001 |
| Kiribati | 1990.68  (1737.14 to 2261.29) | 2205.95 (1915.63 to 2546.3) | -0.17 (-0.2 to -0.14) | <0.001 | 64.8  (41.18 to 97.92) | 70.6 (44.62 to 107.03) | -0.2 (-0.23 to -0.16) | <0.001 |
| Kuwait | 92390.18  (82137.33 to 103687.52) | 2236.35 (1989.54 to 2494.79) | -0.29 (-0.3 to -0.27) | <0.001 | 3617.47  (2433.91 to 5329.05) | 89.43 (61.74 to 130.49) | -0.44 (-0.45 to -0.43) | <0.001 |
| Kyrgyzstan | 87343.36  (76921.51 to 98267.27) | 1460.19 (1283.61 to 1642.02) | -0.11 (-0.13 to -0.09) | <0.001 | 3195.09  (2099.82 to 4759.43) | 53.87 (35.72 to 79.63) | -0.1 (-0.12 to -0.08) | <0.001 |
| Lao People's Democratic Republic | 94507.8  (83462.16 to 104774.55) | 1534.45 (1355.25 to 1718.62) | -0.23 (-0.25 to -0.21) | <0.001 | 3494.04  (2302.79 to 5176.87) | 58.07 (39.02 to 85.36) | -0.38 (-0.42 to -0.34) | <0.001 |
| Latvia | 50732.33  (43595.16 to 57557.32) | 1820.13 (1608.39 to 2053.75) | -0.12 (-0.13 to -0.1) | <0.001 | 1756.78  (1159.4 to 2593.22) | 63.19 (41.55 to 94.56) | -0.15 (-0.16 to -0.14) | <0.001 |
| Lebanon | 143556.66  (127899.34 to 160873.37) | 2523.63 (2245.78 to 2826.2) | -0.44 (-0.46 to -0.42) | <0.001 | 5825.56  (4016.32 to 8329.05) | 101.83 (70.36 to 145.92) | -0.58 (-0.61 to -0.55) | <0.001 |
| Lesotho | 23590.51  (20997.65 to 26311.37) | 1622.94 (1440.93 to 1822.49) | -0.21 (-0.22 to -0.21) | <0.001 | 976.15  (671.07 to 1418.11) | 69.85 (49.33 to 98.4) | -0.4 (-0.42 to -0.38) | <0.001 |
| Liberia | 47407.17  (41895.69 to 52948.36) | 1277.72 (1132.42 to 1447.28) | -0.35 (-0.37 to -0.33) | <0.001 | 2005.6  (1374.66 to 2896.47) | 58.83 (41.59 to 81.87) | -0.53 (-0.56 to -0.51) | <0.001 |
| Libya | 152672.29  (134614.84 to 170012.67) | 2398.4 (2109.72 to 2675.91) | -0.3 (-0.32 to -0.29) | <0.001 | 5783.41  (3924.15 to 8473.88) | 91.86 (62.95 to 132.69) | -0.47 (-0.5 to -0.44) | <0.001 |
| Lithuania | 73821.64  (64056.17 to 84660.81) | 1807.97 (1603.17 to 2022.31) | -0.12 (-0.13 to -0.12) | <0.001 | 2550.01  (1681.17 to 3763.96) | 62.63 (40.88 to 92.62) | -0.16 (-0.18 to -0.14) | <0.001 |
| Luxembourg | 10020.62  (8885.72 to 11010.54) | 1403.43 (1256.6 to 1548.3) | -0.1 (-0.1 to -0.09) | <0.001 | 389.33  (271.89 to 568.75) | 52.49 (35.93 to 77.42) | -0.16 (-0.18 to -0.15) | <0.001 |
| Madagascar | 248179.37  (216271.06 to 279712.55) | 1398.05 (1231.57 to 1584.53) | -0.48 (-0.5 to -0.47) | <0.001 | 10766.93  (7444.56 to 15465.06) | 64.94 (46.05 to 90.88) | -0.63 (-0.65 to -0.61) | <0.001 |
| Malawi | 188109.11  (166719.3 to 208992.59) | 1580.99 (1404.12 to 1763.83) | -0.43 (-0.45 to -0.41) | <0.001 | 8499.62  (5953.45 to 12000.35) | 78.61 (56.68 to 109.63) | -0.62 (-0.65 to -0.58) | <0.001 |
| Malaysia | 445424.53  (393107.25 to 499633.53) | 1437.44 (1265.24 to 1608.99) | -0.3 (-0.31 to -0.28) | <0.001 | 18569.58  (12662.2 to 27013.78) | 60.43 (41.25 to 87.68) | -0.57 (-0.6 to -0.53) | <0.001 |
| Maldives | 8412.14  (7435.94 to 9391.34) | 1940.29 (1716.17 to 2167.36) | -0.36 (-0.41 to -0.3) | <0.001 | 312.7  (210.41 to 464.85) | 73.25 (49.37 to 107.75) | -0.55 (-0.6 to -0.5) | <0.001 |
| Mali | 192059.39  (168694.36 to 215454.13) | 1241.12 (1096.13 to 1399.64) | 0.8 (0.73 to 0.88) | <0.001 | 8138.14  (5561.26 to 11647.59) | 56.76 (39.76 to 80.22) | 0.71 (0.66 to 0.75) | <0.001 |
| Malta | 7460.71  (6628.02 to 8267.51) | 1415.54 (1269.94 to 1571.04) | -0.09 (-0.1 to -0.07) | <0.001 | 298.85  (207.9 to 427.2) | 53.21 (36.04 to 77.98) | -0.17 (-0.18 to -0.16) | <0.001 |
| Marshall Islands | 947.82  (822.24 to 1083.27) | 2186.34 (1874.68 to 2512.18) | -0.2 (-0.23 to -0.16) | <0.001 | 30.62  (19.34 to 47) | 69.44 (43.75 to 106.43) | -0.27 (-0.3 to -0.24) | <0.001 |
| Mauritania | 42837.28  (36919.71 to 48136.25) | 1398.15 (1214.79 to 1603.24) | -0.34 (-0.36 to -0.32) | <0.001 | 1902.39  (1332.93 to 2679.91) | 65.76 (47.02 to 91.23) | -0.51 (-0.52 to -0.5) | <0.001 |
| Mauritius | 28195.47  (24420.21 to 32113.69) | 1863.61 (1634.22 to 2086.98) | -0.32 (-0.34 to -0.3) | <0.001 | 1086.9  (734.91 to 1590.65) | 70.09 (47.39 to 103.12) | -0.5 (-0.52 to -0.47) | <0.001 |
| Mexico | 2854336.69  (2539532.41 to 3163240.09) | 2166.49 (1922.17 to 2397.01) | -0.32 (-0.32 to -0.3) | <0.001 | 123073.49  (85565.92 to 177017.47) | 93.74 (65.42 to 134.43) | -0.47 (-0.49 to -0.45) | <0.001 |
| Micronesia (Federated States of) | 1881.93  (1651.89 to 2179.17) | 2187.09 (1916.71 to 2511.13) | -0.19 (-0.2 to -0.17) | <0.001 | 61.01  (38.91 to 94.33) | 69.92 (44.45 to 108.37) | -0.25 (-0.28 to -0.22) | <0.001 |
| Monaco | 662.75  (589.98 to 738.73) | 1400.53 (1258.16 to 1544.86) | -0.06 (-0.07 to -0.05) | <0.001 | 26.81  (18.46 to 38.3) | 52.41 (35.04 to 77.17) | -0.11 (-0.13 to -0.09) | <0.001 |
| Mongolia | 55492.05  (48901.08 to 62213.06) | 1912.99 (1686.4 to 2154.62) | -0.2 (-0.22 to -0.18) | <0.001 | 2144.41  (1438.85 to 3166.08) | 75.48 (51.37 to 110.53) | -0.26 (-0.29 to -0.24) | <0.001 |
| Montenegro | 9803.11  (8519.84 to 11265.18) | 1240.41 (1091.58 to 1398.44) | -0.04 (-0.06 to -0.01) | 0.01 | 346.46  (226.71 to 513.15) | 43.75 (28.99 to 64.98) | -0.04 (-0.07 to -0.02) | <0.001 |
| Morocco | 693941.24  (618529.55 to 775830.51) | 1888.13 (1680.21 to 2107.85) | -0.12 (-0.13 to -0.1) | <0.001 | 27824.22  (18973.93 to 40906.5) | 76.14 (52.3 to 111.27) | -0.26 (-0.27 to -0.24) | <0.001 |
| Mozambique | 213935.59  (188495.67 to 239798.38) | 1059.81 (948.86 to 1192.2) | 0.11 (0.07 to 0.14) | <0.001 | 9783.55  (6795.27 to 14028.66) | 54 (39.4 to 73.62) | -0.11 (-0.13 to -0.08) | <0.001 |
| Myanmar | 1159735.95  (1026868.66 to 1298607.4) | 2137.77 (1887.89 to 2389.26) | -0.32 (-0.33 to -0.3) | <0.001 | 50432.05  (34292.53 to 73014.86) | 94.4 (65.03 to 135.85) | -0.6 (-0.62 to -0.57) | <0.001 |
| Namibia | 29929.17  (26488.58 to 33395.41) | 1594.62 (1414.64 to 1792.3) | -0.2 (-0.2 to -0.19) | <0.001 | 1233.18  (843.92 to 1795.78) | 68.32 (47.81 to 97.6) | -0.36 (-0.38 to -0.33) | <0.001 |
| Nauru | 166.39  (145.41 to 188.47) | 2184.34 (1882.34 to 2505.2) | -0.17 (-0.2 to -0.13) | <0.001 | 5.4  (3.39 to 8.27) | 69.62 (43.86 to 106.54) | -0.23 (-0.25 to -0.2) | <0.001 |
| Nepal | 684407.93  (606222.82 to 770522.75) | 2586.72 (2275.37 to 2908.46) | 0.03 (-0.03 to 0.1) | 0.327 | 29030.66  (20329.64 to 41572.55) | 112.56 (79.94 to 159.79) | -0.18 (-0.26 to -0.1) | <0.001 |
| Netherlands | 259691.31  (231236.71 to 286149.59) | 1286.62 (1149.94 to 1422.48) | -0.07 (-0.07 to -0.06) | <0.001 | 10174.65  (7101.94 to 14792.43) | 47.97 (32.23 to 70.62) | -0.12 (-0.14 to -0.11) | <0.001 |
| New Zealand | 79111.2  (70843.44 to 87602.68) | 1434.7 (1283.97 to 1594.9) | -0.06 (-0.07 to -0.06) | <0.001 | 2971.18  (2002.08 to 4348.89) | 52.19 (33.95 to 77.1) | -0.1 (-0.12 to -0.08) | <0.001 |
| Nicaragua | 142881.75  (128214.6 to 158227.71) | 2314.7 (2070.85 to 2561.91) | -0.24 (-0.28 to -0.21) | <0.001 | 5712.24  (3932.77 to 8377.71) | 94.95 (66.8 to 138.58) | -0.41 (-0.43 to -0.38) | <0.001 |
| Niger | 209907.24  (184872.83 to 234790.57) | 1408.49 (1252.99 to 1586.47) | 1.09 (1.04 to 1.15) | <0.001 | 9327.18  (6413.71 to 13304.29) | 69.58 (49.92 to 97.68) | 0.97 (0.9 to 1.04) | <0.001 |
| Nigeria | 2292028.41  (2040386.24 to 2560000.49) | 1295.64 (1151.57 to 1447.81) | 0.03 (0.01 to 0.04) | 0.001 | 98225.23  (67354.88 to 142010.95) | 60.37 (42.46 to 85.49) | -0.05 (-0.11 to 0.02) | 0.176 |
| Niue | 43.58  (37.62 to 50.56) | 2143.14 (1857.91 to 2458.51) | -0.22 (-0.25 to -0.18) | <0.001 | 1.38  (0.86 to 2.09) | 67.99 (42.45 to 104.15) | -0.28 (-0.3 to -0.26) | <0.001 |
| North Macedonia | 33966.44  (29417.01 to 38899.43) | 1238.72 (1091.6 to 1394.35) | -0.05 (-0.07 to -0.03) | <0.001 | 1198.59  (791.61 to 1789.96) | 43.67 (29 to 65.26) | -0.07 (-0.1 to -0.03) | <0.001 |
| Northern Mariana Islands | 1152.79  (991.46 to 1350.99) | 2145.78 (1851.19 to 2459.66) | -0.15 (-0.19 to -0.12) | <0.001 | 37.18  (23.08 to 56.14) | 68.79 (42.63 to 103.44) | -0.2 (-0.22 to -0.17) | <0.001 |
| Norway | 88480.54  (79370.44 to 97574.07) | 1441.12 (1288.97 to 1588.67) | 0 (-0.06 to 0.06) | 0.96 | 3400.58  (2343.32 to 4964.03) | 53.18 (35.92 to 78.57) | -0.05 (-0.08 to -0.02) | 0.002 |
| Oman | 157520.41  (139011.44 to 175996.6) | 4007.19 (3604.72 to 4429.02) | 0.49 (0.46 to 0.51) | <0.001 | 5961.85  (3913.54 to 8767.16) | 158.56 (108.57 to 231.4) | 0.48 (0.45 to 0.52) | <0.001 |
| Pakistan | 5118781.09  (4533868.54 to 5732107.19) | 3003.8 (2645.04 to 3432.35) | -0.15 (-0.17 to -0.12) | <0.001 | 203386.02  (137860.27 to 298389.56) | 123.3 (85.14 to 177.46) | -0.16 (-0.18 to -0.14) | <0.001 |
| Palau | 475.97  (409.1 to 563.06) | 2136.83 (1848.76 to 2457.5) | -0.2 (-0.24 to -0.16) | <0.001 | 15.13  (9.46 to 22.89) | 67.64 (42.85 to 101.82) | -0.26 (-0.29 to -0.22) | <0.001 |
| Palestine | 91908.3  (81896.15 to 103171.94) | 2089.07 (1855.84 to 2329.77) | -0.33 (-0.38 to -0.28) | <0.001 | 3357.6  (2225.61 to 4986.29) | 79.43 (53.95 to 116.46) | -0.46 (-0.5 to -0.43) | <0.001 |
| Panama | 106846.76  (96242.73 to 118503.23) | 2443.84 (2202.24 to 2705.23) | -0.13 (-0.17 to -0.08) | <0.001 | 4261.63  (2926.2 to 6201.56) | 97.33 (66.73 to 141.86) | -0.27 (-0.3 to -0.24) | <0.001 |
| Papua New Guinea | 194835.18  (170505.02 to 219645.77) | 2603.95 (2245.76 to 2981.19) | -0.14 (-0.19 to -0.1) | <0.001 | 6216.17  (3951.67 to 9461.65) | 82.08 (51.74 to 124.58) | -0.16 (-0.2 to -0.12) | <0.001 |
| Paraguay | 232756.67  (211354.16 to 254727.78) | 3407.02 (3100.74 to 3724.79) | -0.02 (-0.04 to 0) | 0.023 | 8304  (5479.7 to 12273.15) | 122.24 (81.46 to 180.6) | -0.12 (-0.14 to -0.1) | <0.001 |
| Peru | 1145911.21  (1027842.98 to 1273605.23) | 3196.68 (2875.97 to 3553.96) | -0.06 (-0.09 to -0.03) | <0.001 | 44612.03  (30352.47 to 64728.33) | 124.86 (85.14 to 180.98) | -0.26 (-0.28 to -0.23) | <0.001 |
| Philippines | 2156181.03  (1912948.53 to 2405894.47) | 2131.36 (1886.79 to 2385.18) | -0.12 (-0.16 to -0.09) | <0.001 | 77856.44  (51743.76 to 117261.82) | 77.48 (52.02 to 116.38) | -0.18 (-0.21 to -0.16) | <0.001 |
| Poland | 689857.99  (605921.55 to 784583.11) | 1305.32 (1157.68 to 1458.01) | -0.07 (-0.1 to -0.04) | <0.001 | 24399.98  (16373.19 to 35701.35) | 46.04 (30.76 to 68.01) | -0.05 (-0.09 to -0.01) | 0.007 |
| Portugal | 184534.66  (164895.42 to 206185.88) | 1418.73 (1267.1 to 1574.62) | -0.11 (-0.13 to -0.1) | <0.001 | 7465.04  (5241.48 to 10762.44) | 53.45 (35.77 to 79.14) | -0.21 (-0.22 to -0.19) | <0.001 |
| Puerto Rico | 62141.2  (54599.84 to 70311.56) | 1498.48 (1330.35 to 1666.24) | -0.18 (-0.19 to -0.17) | <0.001 | 2428.2  (1681.29 to 3527.45) | 56.34 (38.15 to 83.55) | -0.28 (-0.3 to -0.26) | <0.001 |
| Qatar | 54199.85  (47305.56 to 61356.23) | 2316.18 (2064.47 to 2571.23) | -0.34 (-0.36 to -0.33) | <0.001 | 2101.16  (1386.88 to 3101.67) | 93.13 (64.04 to 134.68) | -0.53 (-0.55 to -0.5) | <0.001 |
| Republic of Korea | 783709.12  (697275.22 to 873175.09) | 1337.86 (1195.49 to 1478.5) | -0.08 (-0.1 to -0.07) | <0.001 | 30999.87  (21407.65 to 44388.48) | 50.73 (34.35 to 74.56) | -0.16 (-0.19 to -0.13) | <0.001 |
| Republic of Moldova | 117463.64  (104081.34 to 131383.11) | 2444.62 (2172.42 to 2697.55) | 0.17 (0.15 to 0.19) | <0.001 | 3901.3  (2567.21 to 5912.36) | 81.96 (53.72 to 123.42) | 0.12 (0.1 to 0.14) | <0.001 |
| Romania | 339985.34  (292420.12 to 390730.39) | 1250.29 (1101.22 to 1408.74) | -0.04 (-0.06 to -0.02) | <0.001 | 12148.03  (8205.92 to 17674.46) | 44.43 (29.73 to 65.23) | -0.02 (-0.04 to 0.01) | 0.19 |
| Russian Federation | 4354563.4  (3866227.18 to 4930192.87) | 2311.39 (2068.77 to 2573.75) | -0.11 (-0.13 to -0.09) | <0.001 | 157995.16  (105793.67 to 233255.75) | 83.26 (55.94 to 123.39) | -0.17 (-0.18 to -0.17) | <0.001 |
| Rwanda | 99360.33  (87868.39 to 111235.96) | 1099.97 (981.92 to 1238.91) | -0.49 (-0.5 to -0.47) | <0.001 | 4537.31  (3154.75 to 6413.75) | 53.44 (38.11 to 74.19) | -0.72 (-0.73 to -0.7) | <0.001 |
| Saint Kitts and Nevis | 917.67  (809.98 to 1041.96) | 1424.67 (1260.28 to 1594.16) | -0.21 (-0.22 to -0.2) | <0.001 | 34.84  (23.34 to 51.42) | 53.47 (35.94 to 78.75) | -0.32 (-0.34 to -0.31) | <0.001 |
| Saint Lucia | 2854.9  (2521.17 to 3225.6) | 1417.5 (1256.55 to 1591.06) | -0.22 (-0.22 to -0.21) | <0.001 | 109.87  (74.73 to 160.56) | 53.49 (36.42 to 78.83) | -0.33 (-0.35 to -0.3) | <0.001 |
| Saint Vincent and the Grenadines | 1780.58  (1590.56 to 1999.33) | 1433.73 (1280.98 to 1602.14) | -0.21 (-0.23 to -0.2) | <0.001 | 68.4  (46.06 to 100.1) | 54.3 (36.63 to 80.39) | -0.33 (-0.36 to -0.31) | <0.001 |
| Samoa | 3713.68  (3225.06 to 4241.1) | 2191.08 (1895.33 to 2530.17) | -0.17 (-0.24 to -0.11) | <0.001 | 119.61  (76.09 to 179.98) | 69.87 (44.16 to 107.04) | -0.23 (-0.29 to -0.16) | <0.001 |
| San Marino | 559.3  (495.17 to 623.81) | 1400.13 (1249.29 to 1554.78) | -0.06 (-0.07 to -0.05) | <0.001 | 22.39  (15.65 to 31.94) | 52.5 (35.48 to 78) | -0.11 (-0.12 to -0.1) | <0.001 |
| Sao Tome and Principe | 2279.6  (2016.86 to 2552.56) | 1421.54 (1243.14 to 1612.26) | -0.36 (-0.36 to -0.35) | <0.001 | 100.46  (69.6 to 141.51) | 66.4 (46.81 to 91.77) | -0.57 (-0.59 to -0.55) | <0.001 |
| Saudi Arabia | 941424.51  (865971.68 to 1026714.94) | 3015.33 (2762.64 to 3293.38) | -0.52 (-0.54 to -0.5) | <0.001 | 38194.68  (26129.6 to 54353.58) | 125.68 (86.99 to 178.6) | -0.74 (-0.77 to -0.72) | <0.001 |
| Senegal | 156321.79  (138766.86 to 174908.15) | 1432.35 (1266.22 to 1617.32) | -0.27 (-0.29 to -0.25) | <0.001 | 7837.47  (5635.2 to 10741.97) | 77.68 (57.31 to 106.33) | -0.53 (-0.55 to -0.52) | <0.001 |
| Serbia | 152485.73  (132768.22 to 174269.75) | 1240.19 (1093.16 to 1401.63) | -0.05 (-0.06 to -0.04) | <0.001 | 5404.45  (3659.26 to 7864.41) | 43.79 (29.69 to 64.39) | -0.06 (-0.09 to -0.02) | 0.001 |
| Seychelles | 2104.38  (1844.62 to 2404.78) | 1884.62 (1662.13 to 2126.41) | -0.27 (-0.3 to -0.24) | <0.001 | 80.32  (53.89 to 116.82) | 71.3 (48.45 to 104.37) | -0.45 (-0.48 to -0.42) | <0.001 |
| Sierra Leone | 81030.22  (72269.45 to 90261.03) | 1368.06 (1226.01 to 1512.26) | -0.19 (-0.22 to -0.16) | <0.001 | 3627.3  (2556.84 to 5166.11) | 67.25 (48.67 to 92.51) | -0.34 (-0.36 to -0.32) | <0.001 |
| Singapore | 104061.75  (92484.97 to 116108.29) | 1703.83 (1528.47 to 1885.94) | -0.09 (-0.11 to -0.07) | <0.001 | 4035.03  (2727.25 to 5977.27) | 63.82 (42.43 to 95.17) | -0.15 (-0.16 to -0.13) | <0.001 |
| Slovakia | 91298.61  (79435.63 to 105003.24) | 1246.56 (1101.12 to 1401.27) | -0.04 (-0.07 to -0.01) | 0.008 | 3233.33  (2166.49 to 4798.32) | 44.02 (29.33 to 66.08) | -0.03 (-0.06 to 0) | 0.072 |
| Slovenia | 38130.43  (32909.9 to 43691.3) | 1235.01 (1086.48 to 1399.57) | -0.08 (-0.1 to -0.05) | <0.001 | 1347.2  (893.94 to 1976.92) | 43.49 (28.65 to 64.89) | -0.05 (-0.09 to -0.02) | 0.005 |
| Solomon Islands | 10396.56  (9108.17 to 11676.89) | 2239.38 (1939.9 to 2568.05) | -0.11 (-0.14 to -0.07) | <0.001 | 341.77  (217.18 to 517.09) | 72.29 (45.31 to 109.74) | -0.15 (-0.17 to -0.12) | <0.001 |
| Somalia | 112305.29  (98051.11 to 127040.92) | 754.16 (682.41 to 822.67) | -0.31 (-0.34 to -0.29) | <0.001 | 4816.55  (3300.9 to 6912.61) | 36.92 (26.54 to 50.89) | -0.43 (-0.45 to -0.4) | <0.001 |
| South Africa | 772493.66  (683177.97 to 863879.29) | 1487.33 (1315.29 to 1667.61) | 0.02 (-0.06 to 0.11) | 0.608 | 32498.27  (22827.73 to 46583.52) | 63.59 (45.18 to 89.92) | -0.07 (-0.14 to -0.01) | 0.016 |
| South Sudan | 76001.88  (65553.46 to 87234.29) | 884.78 (780.82 to 991.63) | -0.35 (-0.4 to -0.3) | <0.001 | 2943.23  (1944.01 to 4318.7) | 37.67 (26.29 to 54.01) | -0.57 (-0.61 to -0.53) | <0.001 |
| Spain | 1398197.12  (1245931.95 to 1552577.97) | 2596.83 (2333.55 to 2900.07) | 0.03 (0.02 to 0.04) | <0.001 | 58298.19  (40774.47 to 84173.84) | 101.76 (68.6 to 150.7) | -0.05 (-0.06 to -0.04) | <0.001 |
| Sri Lanka | 539244.22  (471092.01 to 609186.26) | 2198.2 (1931.93 to 2457.35) | -0.28 (-0.34 to -0.21) | <0.001 | 20279.99  (13664.57 to 29602.67) | 81.51 (54.68 to 119.95) | -0.44 (-0.51 to -0.38) | <0.001 |
| Sudan | 684154.13  (613557.78 to 760761.66) | 1947.12 (1747.9 to 2146.95) | -0.39 (-0.43 to -0.36) | <0.001 | 29149.61  (20082.55 to 42602.5) | 90.61 (64.23 to 127.23) | -0.66 (-0.69 to -0.63) | <0.001 |
| Suriname | 8296.68  (7322.95 to 9349.45) | 1382.9 (1224.6 to 1553.85) | -0.23 (-0.26 to -0.21) | <0.001 | 308.35  (206.87 to 457.22) | 51.07 (34.24 to 76.79) | -0.35 (-0.39 to -0.31) | <0.001 |
| Sweden | 85457.67  (74283.18 to 95348.25) | 706.37 (613.59 to 797.73) | 0.02 (-0.01 to 0.04) | 0.186 | 3595.35  (2504.75 to 5114.24) | 28.23 (19.14 to 40.94) | -0.02 (-0.04 to 0) | 0.028 |
| Switzerland | 146662.71  (131098.22 to 163198.44) | 1401.29 (1249.78 to 1556.86) | -0.05 (-0.06 to -0.04) | <0.001 | 5758.69  (4014.58 to 8304.91) | 52.21 (34.83 to 77.51) | -0.09 (-0.12 to -0.06) | <0.001 |
| Syrian Arab Republic | 338585.76  (302036.95 to 379840.27) | 2400.03 (2154.81 to 2675.2) | -0.32 (-0.32 to -0.31) | <0.001 | 14002.73  (9736.93 to 20069.62) | 99.73 (69.75 to 142.83) | -0.5 (-0.52 to -0.48) | <0.001 |
| Taiwan (Province of China) | 380710.59  (330532.07 to 439387.98) | 1091.77 (965.07 to 1234.83) | -0.34 (-0.38 to -0.3) | <0.001 | 12528.95  (8196.6 to 18918.52) | 35.98 (23.44 to 54.17) | -0.54 (-0.58 to -0.49) | <0.001 |
| Tajikistan | 145438.9  (128718.71 to 162941.04) | 1743.9 (1541.72 to 1951.83) | -0.11 (-0.12 to -0.1) | <0.001 | 5402.63  (3577.14 to 7991.19) | 66.27 (44.71 to 98.07) | -0.12 (-0.14 to -0.1) | <0.001 |
| Thailand | 1268260.61  (1147156.43 to 1396533.45) | 1666.92 (1492.96 to 1854.36) | -0.48 (-0.54 to -0.42) | <0.001 | 47613.99  (32358.21 to 69131.88) | 60.06 (39.62 to 88.93) | -0.58 (-0.65 to -0.51) | <0.001 |
| Timor-Leste | 31151.67  (27664.48 to 34804.78) | 2760.87 (2461.47 to 3072.01) | -0.29 (-0.41 to -0.17) | <0.001 | 1102.9  (722.23 to 1628.54) | 99.76 (66.68 to 148.14) | -0.42 (-0.52 to -0.32) | <0.001 |
| Togo | 84407.85  (73924.03 to 94543.86) | 1439.11 (1266.76 to 1649.15) | 0.32 (0.29 to 0.36) | <0.001 | 3714.3  (2572.64 to 5303.46) | 67.97 (48.33 to 95.59) | 0.29 (0.25 to 0.32) | <0.001 |
| Tokelau | 31.78  (27.31 to 36.44) | 2176.66 (1872.32 to 2492.89) | -0.23 (-0.29 to -0.18) | <0.001 | 1.01  (0.63 to 1.54) | 69.4 (43.6 to 105.78) | -0.31 (-0.37 to -0.25) | <0.001 |
| Tonga | 1671.75  (1467.41 to 1896.59) | 1896.25 (1649.78 to 2165.24) | -0.26 (-0.29 to -0.23) | <0.001 | 53.99  (34.04 to 81.22) | 60.87 (38.07 to 91.84) | -0.37 (-0.46 to -0.28) | <0.001 |
| Trinidad and Tobago | 24140.45  (21943.53 to 26484.97) | 1525.2 (1381.93 to 1673.9) | -0.25 (-0.26 to -0.24) | <0.001 | 902.75  (615.93 to 1324.77) | 56.07 (37.99 to 83) | -0.37 (-0.4 to -0.34) | <0.001 |
| Tunisia | 224767.23  (197124.63 to 255225.74) | 1795.66 (1589.38 to 2023.15) | -0.27 (-0.28 to -0.26) | <0.001 | 9331.36  (6487.82 to 13400.87) | 73.83 (51.44 to 107.07) | -0.48 (-0.5 to -0.48) | <0.001 |
| Türkiye | 1482165.75  (1309604.01 to 1672287.51) | 1676.01 (1487.99 to 1872.69) | 0.01 (-0.02 to 0.05) | 0.378 | 59930.88  (41274.82 to 87023.2) | 67.31 (46.39 to 97.47) | -0.18 (-0.21 to -0.15) | <0.001 |
| Turkmenistan | 84098.91  (74000.92 to 94264.86) | 1704.32 (1498.76 to 1906.04) | -0.23 (-0.24 to -0.22) | <0.001 | 3052.56  (2003.69 to 4584.55) | 62.38 (41.12 to 93.4) | -0.24 (-0.27 to -0.22) | <0.001 |
| Tuvalu | 248.83  (217.36 to 285.9) | 2209.02 (1929.1 to 2532.33) | -0.21 (-0.22 to -0.2) | <0.001 | 8.03  (5.06 to 12.17) | 70.81 (44.71 to 107.22) | -0.28 (-0.3 to -0.26) | <0.001 |
| Uganda | 235450.77  (205420.78 to 265562.75) | 790.76 (702.69 to 884.3) | 0.67 (0.62 to 0.72) | <0.001 | 10336.69  (7091.91 to 14828.82) | 38.61 (27.58 to 53.34) | 0.41 (0.32 to 0.49) | <0.001 |
| Ukraine | 1142047  (992562.2 to 1300423.34) | 1915.71 (1702.61 to 2141.97) | -0.09 (-0.1 to -0.08) | <0.001 | 39803.18  (26044.39 to 58377.48) | 67 (44.33 to 100.25) | -0.11 (-0.12 to -0.11) | <0.001 |
| United Arab Emirates | 180164.9  (157233.91 to 207119.02) | 2260 (2004.45 to 2521.72) | -0.34 (-0.34 to -0.33) | <0.001 | 7184.74  (4740.57 to 10538.71) | 91.91 (64.29 to 133.51) | -0.5 (-0.52 to -0.48) | <0.001 |
| United Kingdom | 1296024.09  (1166949.76 to 1426595.96) | 1671.53 (1507.47 to 1851.53) | -0.08 (-0.09 to -0.07) | <0.001 | 49715.48  (34416.68 to 72101.81) | 61.32 (41.05 to 90.63) | -0.15 (-0.16 to -0.14) | <0.001 |
| United Republic of Tanzania | 505147.43  (447809.51 to 562552.95) | 1249.83 (1112.74 to 1391.15) | -0.41 (-0.44 to -0.37) | <0.001 | 24126.53  (17135.39 to 33601.99) | 65.11 (47.27 to 88.47) | -0.45 (-0.49 to -0.41) | <0.001 |
| United States of America | 3867960.12  (3446162.13 to 4247429.96) | 1073.85 (956.17 to 1192.41) | 0.05 (0.03 to 0.07) | <0.001 | 149609.42  (102963.92 to 216342.43) | 39.94 (26.77 to 58.7) | -0.02 (-0.04 to 0) | 0.012 |
| United States Virgin Islands | 1623.76  (1423.05 to 1845.95) | 1499.88 (1332.06 to 1679) | -0.19 (-0.22 to -0.15) | <0.001 | 63.89  (44.14 to 93.72) | 56.69 (38.51 to 84.69) | -0.28 (-0.31 to -0.24) | <0.001 |
| Uruguay | 66615.03  (59519.13 to 73984.68) | 1725.78 (1538.55 to 1906.85) | -0.12 (-0.15 to -0.09) | <0.001 | 2451.36  (1635.47 to 3610.37) | 61.51 (40.24 to 92.54) | -0.16 (-0.18 to -0.14) | <0.001 |
| Uzbekistan | 548650.46  (483999.49 to 620715.36) | 1745.91 (1543.09 to 1957.3) | -0.15 (-0.16 to -0.14) | <0.001 | 20416.14  (13533.83 to 30697.84) | 65.65 (44.11 to 98.09) | -0.19 (-0.2 to -0.18) | <0.001 |
| Vanuatu | 4449.64  (3882.21 to 5067.68) | 1990.39 (1716.32 to 2306.27) | -0.12 (-0.14 to -0.11) | <0.001 | 144.31  (91.21 to 219.46) | 63.63 (39.5 to 96.7) | -0.15 (-0.18 to -0.13) | <0.001 |
| Venezuela (Bolivarian Republic of) | 608866.07  (543508.49 to 678982.9) | 2165.67 (1948.51 to 2389.56) | -0.11 (-0.13 to -0.1) | <0.001 | 25796.3  (17824.14 to 36824.22) | 91.05 (62.72 to 130.08) | -0.22 (-0.26 to -0.18) | <0.001 |
| Viet Nam | 1615453.93  (1461234.6 to 1771171.51) | 1624.04 (1472.86 to 1780.55) | -0.26 (-0.34 to -0.18) | <0.001 | 68703.17  (47515.81 to 99588.14) | 68.95 (48.03 to 100.28) | -0.52 (-0.57 to -0.47) | <0.001 |
| Yemen | 535914.14  (468338.52 to 606778.53) | 2077.03 (1815.39 to 2343.19) | 0.51 (0.45 to 0.57) | <0.001 | 21765.11  (14671.27 to 31952.78) | 89.86 (62.51 to 128.74) | 0.48 (0.45 to 0.52) | <0.001 |
| Zambia | 141017.8  (123508.2 to 159508.33) | 1178.61 (1030.42 to 1339.99) | -0.4 (-0.44 to -0.37) | <0.001 | 5304.74  (3559.02 to 7960.51) | 46.72 (32.01 to 67.74) | -0.57 (-0.61 to -0.52) | <0.001 |
| Zimbabwe | 190961.9  (170506.06 to 212251.69) | 1785.58 (1582.57 to 1992.12) | -0.2 (-0.25 to -0.16) | <0.001 | 8141.6  (5613.05 to 11949.37) | 82.09 (58.04 to 115.2) | -0.36 (-0.39 to -0.32) | <0.001 |

DALYs, disability adjusted life years; UI, uncertainty interval; SDI, Socio-demographic Index; AAPC: average annual percent change; CI: Confidence Interval.
